# GLS1 Orchestrates Exosome‐Mediated Tumor‐Endothelial Communication to Facilitate Angiogenesis

**DOI:** 10.1002/advs.75510

**Published:** 2026-05-03

**Authors:** Jianqiang Yang, Zhenzhen Fu, Fanghui Chen, Soumya Vijaya Kumar, Fan Yang, Yaochao Zheng, Yunqi Li, Yao Yao, Nabil F. Saba, Yong Teng

**Affiliations:** ^1^ Department of Hematology and Medical Oncology Winship Cancer Institute School of Medicine Emory University Atlanta Georgia USA; ^2^ Regenerative Bioscience Center Department of Animal and Dairy Science University of Georgia Athens Georgia USA; ^3^ Wallace H. Coulter Department of Biomedical Engineering Georgia Institute of Technology & Emory University Atlanta Georgia USA

**Keywords:** angiogenesis, CAV1‐TNC signaling, exosomes, GLS1, HNSC

## Abstract

Glutaminase 1 (GLS1) drives glutaminolysis to support tumor growth and survival, yet its role in the tumor microenvironment remains poorly understood. Here, we demonstrate that GLS1 promotes angiogenesis in head and neck squamous cell carcinoma (HNSCC) via an exosome‐dependent mechanism. In HNSCC xenograft models, genetic silencing of *GLS1* or treatment with CB‐839 markedly reduces intratumoral angiogenesis. Exosomes from *GLS1*‐deficient cells impair endothelial cell migration and tube formation compared with control exosomes. Proteomic analysis reveals a loss of the pro‐angiogenic protein Tenascin C (TNC) in *GLS1*‐deficient exosomes. Mechanistically, loss of *GLS1* interferes with USP1‐mediated deubiquitination of Caveolin‐1 (CAV1), resulting in CAV1 degradation and impaired recruitment of TNC into exosomes. Exosomes deficient in CAV1‐TNC complexes subsequently disrupt integrin‐dependent FAK‐SRC signaling in endothelial cells, inhibiting their angiogenic activity. Collectively, these findings uncover a non‐metabolic role of GLS1 in promoting tumor angiogenesis through exosome‐mediated CAV1‐TNC signaling, suggesting that targeting GLS1 may simultaneously inhibit tumor metabolism and angiogenesis in HNSCC.

## Graphical abstract

This study reveals a previously unappreciated, non‐metabolic function of GLS1 in the control of tumor angiogenesis via exosome‐mediated CAV1‐TNC signaling, indicating that therapeutic targeting of GLS1 may offer a dual benefit by simultaneously suppressing tumor metabolic activity and angiogenic processes in head and neck cancer.

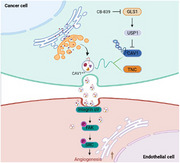

## 1 Introduction

Glutamine metabolism is profoundly reprogrammed during tumorigenesis to support the bioenergetic and biosynthetic demands of rapidly proliferating cancer cells. Glutamine serves as a versatile nutrient that fuels the synthesis of proteins, nucleotides, and lipids, replenishes tricarboxylic acid (TCA) cycle intermediates, and maintains redox homeostasis by contributing to glutathione production [1, 2, 3]. This metabolic dependency, often referred to as “glutamine addiction,” represents a hallmark of cancer cell metabolic reprogramming. Notably, the upregulation of critical glutamine transporters and metabolic enzymes, particularly glutaminase 1 (GLS1), the enzyme responsible for converting glutamine to glutamate, has been strongly associated with tumor progression, therapeutic resistance, and unfavorable clinical outcomes across multiple cancer types [1, 4, 5, 6]. Our previous studies have demonstrated that head and neck squamous cell carcinoma (HNSCC) cells display a marked dependence on glutamine metabolism [1, 7]. Among the enzymes involved, GLS1 plays a pivotal role in sustaining HNSCC cell growth and survival by catalyzing glutamine utilization [7]. Owing to its central function in tumor metabolic reprogramming, GLS1 has emerged as a compelling therapeutic target in multiple cancer types [4, 8, 9]. Nevertheless, the mechanistic basis through which GLS1 influences the interplay between cancer cells and the tumor microenvironment (TME) remains poorly understood and warrants further investigation.

Recent advances in cancer biology have shifted the conceptual framework from a tumor‐centric perspective to a more integrated view that emphasizes the complex and dynamic interactions between cancer cells and their surrounding TME. The TME constitutes a highly heterogeneous and adaptive ecosystem, composed not only of malignant cells but also of diverse non‐malignant stromal cell populations, including cancer‐associated fibroblasts (CAFs), immune cells, and endothelial cells [10, 11]. In addition, non‐cellular components such as the extracellular matrix (ECM), soluble signaling molecules, and extracellular vesicles (EVs), particularly exosomes, play critical roles in shaping tumor behavior [12]. Exosomes, nanosized vesicles with an average diameter of approximately 100 nm, have emerged as pivotal mediators of intercellular communication within the TME [12, 13]. By transferring proteins, lipids, and nucleic acids between cells, exosomes enable tumor cells to reprogram surrounding stromal and immune cells, thereby remodeling the TME to promote tumor progression, angiogenesis, immune evasion, and therapeutic resistance [14]. For example, gallbladder cancer‐derived exosomes enriched with the long noncoding RNA TRPM2‐AS have been shown to activate NOTCH1 signaling in human umbilical vein endothelial cells (HUVECs), thereby enhancing their pro‐angiogenic activity [15]. Likewise, breast cancer cell‐derived exosomes containing miR‐105 disrupt vascular integrity by downregulating ZO‐1, resulting in increased vascular permeability and promoting metastatic dissemination [16]. However, comparatively few studies have investigated how exosome‐associated proteins influence the TME and contribute to cancer progression.

Emerging studies have uncovered that numerous metabolic enzymes exhibit moonlighting functions that actively contribute to cancer initiation and progression. In addition to their canonical biochemical roles, these enzymes engage in diverse cellular processes that drive tumorigenesis, metastasis, and therapeutic resistance. For example, creatine kinase B (CKB) has been identified to function as a protein kinase, phosphorylating and stabilizing glutathione peroxidase 4 (GPX4), which in turn suppresses ferroptosis and facilitates tumor growth in hepatocellular carcinoma [17]. Our previous study demonstrated that enolase 2 (ENO2), a key glycolytic enzyme involved in cancer metabolism, also functions as a moonlighting protein that regulates PKM2 protein stability and nuclear translocation, thereby promoting PKM2‐mediated glycolytic flux and CCND1‐driven cell cycle progression [18]. In this study, we present the first evidence that GLS1, enriched in HNSCC cells, has a noncanonical pro‐angiogenic role that extends beyond its well‐known metabolic function. Specifically, GLS1 promotes angiogenesis through an exosome‐mediated Caveolin‐1 (CAV1)‐Tenascin C (TNC) signaling axis, facilitating communication between tumor and endothelial cells. Given that HNSCC develops within a highly vascularized TME, our findings position GLS1 as a promising dual‐action therapeutic target capable of simultaneously disrupting tumor metabolism and tumor‐induced vascularization in HNSCC.

## 2 Results

### 2.1 Genetic Depletion or Pharmacologic Inhibition of GLS1 Impairs Angiogenesis Through an Exosome‐Dependent Mechanism

**FIGURE 1:**
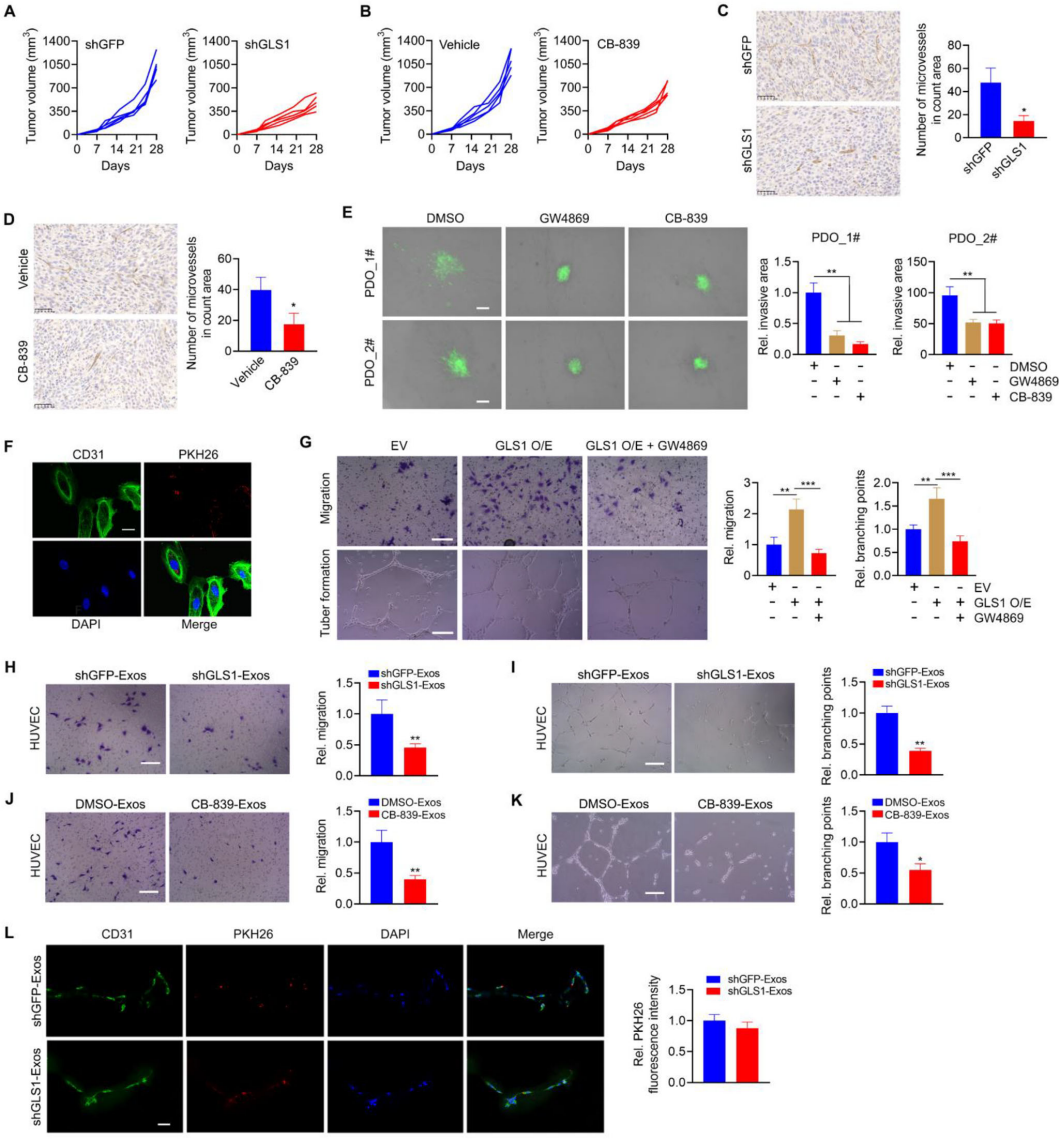
Genetic knockdown or pharmacological inhibition of *GLS1* impairs angiogenesis through an exosome‐mediated mechanism. (A) Effect of *GLS1* knockdown on tumor growth in NSG mice (n = 5 mice/ group). *GLS1* knockdown or control HN12 cells were injected into the buccal mucosa of NSG mice under anesthesia. The experiment was terminated 28 days after cell inoculation. (B) Effect of CB‐839 treatment on tumor growth in NSG mice bearing HN12 tumors (n = 5 mice/ group). CB‐839 was administered by oral gavage twice a day at a dose of 200 mg/kg body weight for 18 days, starting 10 days after HN12 cell inoculation. The experiment was terminated on Day 28. (C) IHC of tumor tissues derived from *GLS1* knockdown or control HN12 xenografts using anti‐CD31 antibody. Quantitative data (n = 5 mice/ group) are presented in the right panel. (D) IHC of tumor tissues derived from HN12 xenografts treated with CB‐839 or vehicle using anti‐CD31 antibody. Quantitative data (n = 5 mice/ group) are presented in the right panel. (E) HUVEC invasion following co‐culture with HNSCC PDOs (PDO_1# and PDO_2#, derived from two individual HNSCC patients) treated with or without 20 µm GW4869 or 5 µm CB‐839 in a 3D agarose cast. HUVECs were labeled with CellTracker Green BODIPY (green) before co‐cultured with PDOs in agarose casts. Scale bar: 100 µm. (F) Confocal microscopy images showing the uptake of HN12 cell‐derived exosomes by HUVECs. HUVECs were stained with anti‐CD31 antibody (green), exosome membranes were labeled with PKH26 (red), and nuclei were counterstained with DAPI (blue). Scale bar: 20 µm. (G) HUVEC migration and tuber formation following a 24‐h co‐culture with conditioned media from *GLS1* overexpressing (GLS1 O/E) or control (EV) HN12 cells, in the presence or absence of 20 µm GW4869. Scale bar: 50 µm. (H) HUVEC migration following a 24‐h transwell incubation with exosomes from *GLS1* knockdown (shGLS1‐Exos) or control (shGFP‐Exos) HN12 cells. Scale bar: 50 µm. (I) HUVEC tube formation following a 24‐h co‐culture with exosomes from *GLS1* knockdown or control HN12 cells. Scale bar: 50 µm. (J) HUVEC migration following a 24‐h transwell incubation with exosomes from HN12 cells treated with 2 µm CB‐839 (CB‐839‐Exos) or DMSO (DMSO‐Exos). Scale bar: 50 µm. (K) HUVEC tube formation following a 24‐h co‐culture with exosomes from HN12 cells treated with 2 µm CB‐839 or DMSO. Scale bar: 50 µm. (L) Confocal microscopy images showing HUVEC uptake of exosomes derived from *GLS1* knockdown or control HN12 cells. Scale bar: 50 µm. HUVECs were stained with anti‐CD31 antibody (green), exosome membranes were labeled with PKH26 (red), and nuclei were counterstained with DAPI (blue). In (C‐E and G‐L), representative images and quantitative data (n = 3) are shown in the left and right panels, respectively. Exosomes were used at a concentration of 100 µg/ml in this study. ^*^ *p* < 0.05; ^**^ *p* < 0.01.

To investigate the role of GLS1 in tumor progression, we established a xenograft model by intramucosal injection of *GLS1* knockdown or control HN12 cells into NSG mice. Genetic silencing of *GLS1* markedly suppressed tumor growth, resulting in smaller and slower‐growing xenografts compared with controls (Figure 1A). Consistently, pharmacological inhibition of GLS1 using CB‐839 led to significant regression of HN12 tumors in mice (Figure 1B). Notably, immunohistochemical (IHC) analysis of the resulting tumor tissues revealed a pronounced reduction in CD31‐positive vasculature following either genetic depletion of *GLS1* or pharmacological inhibition with CB‐839 (Figure 1C,D), supporting the role of GLS1 in promoting tumor angiogenesis. To determine whether GLS1 similarly regulates endothelial cells within an intact, immunocompetent microenvironment, we depleted the mouse *Gls* gene in murine oral cancer MOC2 cells and implanted them into C57BL/6 mice. Consistent with the reduced tumor growth, a marked reduction in CD31‐positive vessels was observed in *Gls* knockdown tumors relative to control tumors (Figure S1), suggesting that tumor‐intrinsic GLS1 signaling contributes to the regulation of tumor angiogenesis in the context of the immune system.

To assess whether tumor‐derived exosomes contribute to GLS1‐mediated tumor angiogenesis, we inhibited exosome biogenesis using a neutral sphingomyelinase inhibitor GW4869. Conditioned media collected from GW4869‐treated or control HN12 cells was used as a chemoattractant in a 24‐h transwell migration assay with HUVECs. Compared with control conditioned media, media from GW4869‐treated cells significantly reduced HUVEC migration (Figure S2). Consistently, in 3D co‐culture assays with HNSCC patient‐derived organoids (PDO_1# and PDO_2#) and HUVECs, treatment of PDOs with GW4869 markedly impaired HUVEC invasion, as indicated by a reduced invasive area (Figure 1E). Immunofluorescence (IF) analysis confirmed the efficient uptake of HN12 cell‐derived exosomes by HUVECs (Figure 1F). To more directly assess whether GLS1 regulates HUVEC function through exosome‐mediated mechanisms, HUVECs were co‐cultured with conditioned media from *GLS1* overexpressing or control HN12 cells. Conditioned media from *GLS1* overexpressing cells markedly enhanced HUVEC migration and tube formation compared with control media (Figure 1G). Notably, these pro‐angiogenic effects were largely abrogated when the conditioned media was generated in the presence of GW4869 (Figure 1G). In contrast, HUVECs co‐cultured with exosomes from *GLS1* knockdown HN12 cells exhibited markedly reduced migratory capacity and tube formation ability compared with those co‐cultured with control exosomes (Figure 1H,I). A similar trend was observed in HUVECs exposed to exosomes from *GLS1* knockdown HN6 cells (Figure S3). While CB‐839 alone did not affect HUVEC proliferation and migration (Figure S4), exosomes isolated from CB‐839‐treated HN12 cells significantly suppressed HUVEC migration and tube formation compared with those isolated from control cells (Figure 1J,K). In PDO‐HUVEC 3D co‐cultures, treatment of PDOs with CB‐839, similar to GW4869, impaired HUVEC invasive capacity (Figure 1F). These findings support the notion that GLS1 modulates endothelial cell motility predominantly through tumor‐derived exosomes.

Interestingly, nanoparticle tracking analysis showed that *GLS1* knockdown did not alter the size distribution of exosomes released from HNSCC cells (Figure S5). Consistent with these findings, IF analysis revealed no significant reduction in the overall number of exosomes internalized by HUVECs following *GLS1* knockdown (Figure 1L), indicating that tumor‐intrinsic GLS1 promotes angiogenesis primarily by altering exosomal cargo composition rather than influencing exosome uptake.

### 2.2 GLS1 Enhances Tumor Angiogenesis Through the Regulation of Exosomal TNC Signaling

**FIGURE 2:**
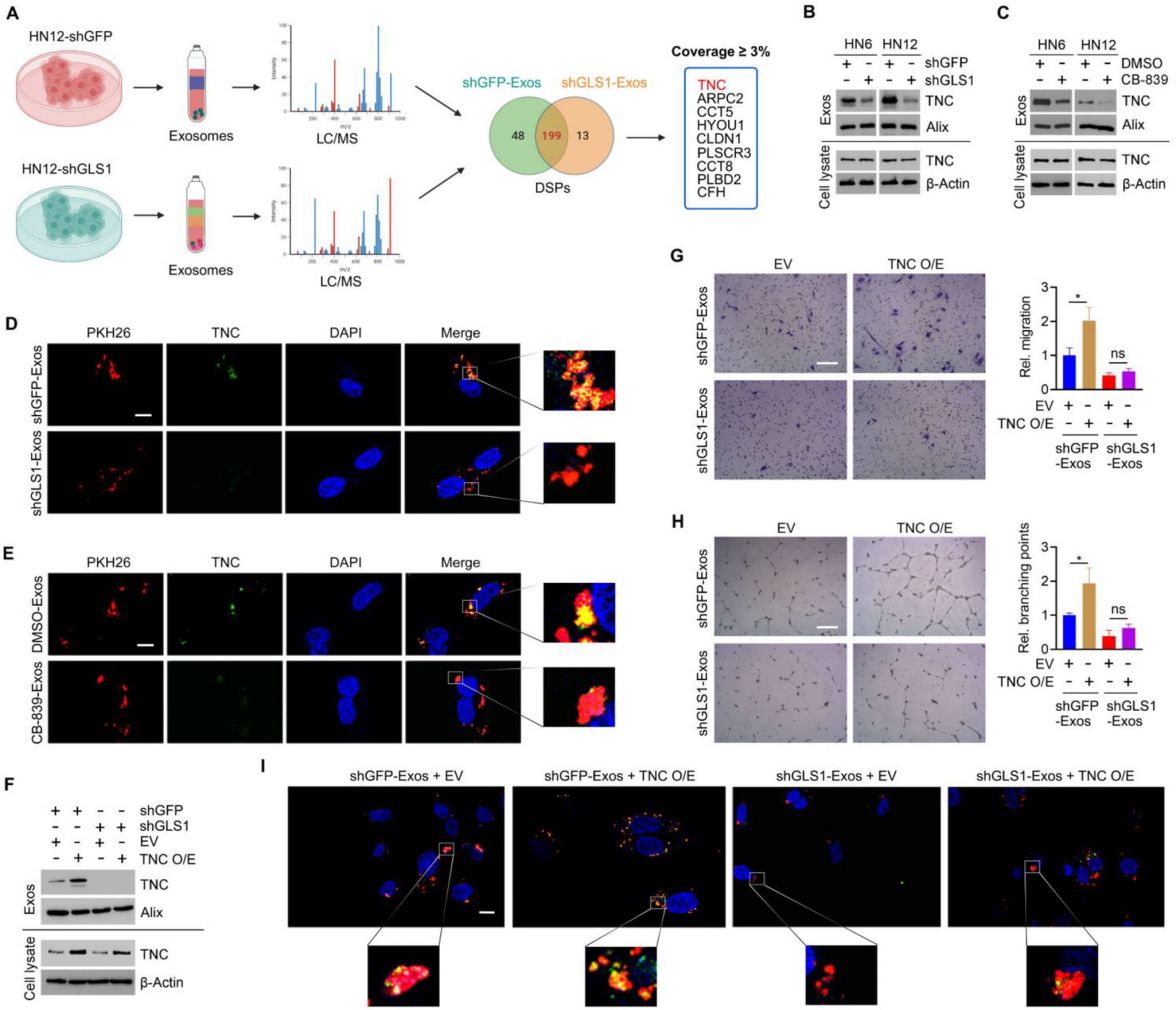
GLS1‐driven exosomal TNC promotes angiogenesis in HNSCC. (A) Schematic illustration of the workflow used to identify differentially secreted proteins (DSPs) in exosomes derived from *GLS1* knockdown and control HN12 cells. Figure created using Biorender (https://biorender.com). (B) Effect of *GLS1* knockdown on TNC protein levels in HN6 and HN12 cells and their derived exosomes determined by Western blot. (C) Effect of CB‐839 treatment on TNC protein levels in HN6 and HN12 cells and their derived exosomes determined by Western blot. (D) Confocal microscopy images showing internalization of exosomal TNC by HUVECs after 24 h of co‐culture with exosomes derived from *GLS1* knockdown or control HN12 cells. Scale bar: 20 µm. (E) Confocal microscopy images showing internalization of exosomal TNC by HUVECs after 24 h of co‐culture with exosomes derived from HN12 cells treated with 2 µm CB‐839 or DMSO. Scale bar: 20 µm. (F) Effect of TNC overexpression (O/E) on TNC protein levels in *GLS1* knockdown or control HN12 cells and their derived exosomes determined by Western blot. (G) HUVEC migration following a 24‐h transwell incubation with exosomes from *GLS1* knockdown or control HN12 cells, with or without TNC overexpression (O/E). (H) HUVEC tube formation following a 24‐h co‐culture with exosomes from *GLS1* knockdown or control HN12 cells, with or without TNC overexpression (O/E). (G, H) Representative images and quantitative data (n = 3) are shown in the left and right panels, respectively. Scale bar: 50 µm. (I) Confocal microscopy images showing internalization of exosomal TNC by HUVECs after 24 h of co‐culture with exosomes derived from *GLS1* knockdown or control HN12 cells, with or without TNC O/E. Scale bar: 20 µm. In (D, E and I), exosomal TNC was stained with anti‐TNC antibody (green), exosome membranes were labeled with PKH26 (red), and nuclei were counterstained with DAPI (blue). Exosomes were used at a concentration of 100 µg/ml in this study. ns: not significant; ^*^ *p* < 0.05; ^**^ *p* < 0.01.

Tumor‐derived exosomes shuttle a complex cargo of proteins, lipids, and nucleic acids and thereby modulate neighboring stromal, immune, and endothelial cells within the TME [12]. To identify GLS1‐dependent exosomal proteins, we isolated exosomes from *GLS1* knockdown and control HN12 cells via differential centrifugation and analyzed their protein cargo using liquid chromatography‐mass spectrometry (LC‐MS). Proteomic profiling revealed 48 differentially secreted proteins (DSPs) that were detected in exosomes from control HN12 cells but absent or below the detection threshold in exosomes derived from *GLS1* knockdown cells (Figure 2A). Among them, TNC was a prominent candidate, exhibiting the highest protein coverage in the LC‐MS dataset (14.54%) (Figure 2A; Figure S6 and Table S1). Western blot analysis confirmed a marked reduction in TNC protein levels in exosomes derived from both HN12 and HN6 cells upon *GLS1* knockdown (Figure 2B). However, no noticeable difference in TNC abundance was observed between *GLS1* knockdown and control HNSCC cells (Figure 2B), or in their corresponding exosome‐depleted supernatants (Figure S7), suggesting that GLS1 primarily influences the selective packaging of TNC into exosomes rather than its expression. Consistently, CB‐839 treatment reduced the abundance of TNC in exosomes but did not alter its intracellular levels (Figure 2C). By combining PKH26 membrane labeling with anti‐TNC immunostaining, we observed a marked reduction in the number of TNC‐positive exosomes from *GLS1* knockdown HN12 cells internalized by HUVECs compared with those from control cells (Figure 2D). CB‐839 treatment elicited a comparable reduction in the uptake of TNC‐positive exosomes by HUVECs (Figure 2E), further supporting the notion that GLS1 regulates the exosome‐mediated transfer of TNC to endothelial cells.

Moreover, overexpression of *TNC* in *GLS1* knockdown HN12 cells increased TNC expression levels; however, this was not accompanied by a corresponding increase in exosomal TNC (Figure 2F). Moreover, co‐culture of HUVECs with exosomes derived from *GLS1* knockdown HN12 cells, regardless of *TNC* overexpression, failed to enhance HUVEC migration or tube formation compared with controls (Figure 2G,H). IF analysis further showed that *GLS1* knockdown HN12 cells with *TNC* overexpression did not restore the uptake of TNC‐positive exosomes by HUVECs (Figure 2I). These findings indicate that GLS1 is essential for the efficient packaging of TNC into exosomes, thereby promoting endothelial cell motility and angiogenesis in HNSCC.

### 2.3 GLS1 Regulates the Exosomal Packaging of TNC Through a CAV1‐Dependent Mechanism

**FIGURE 3:**
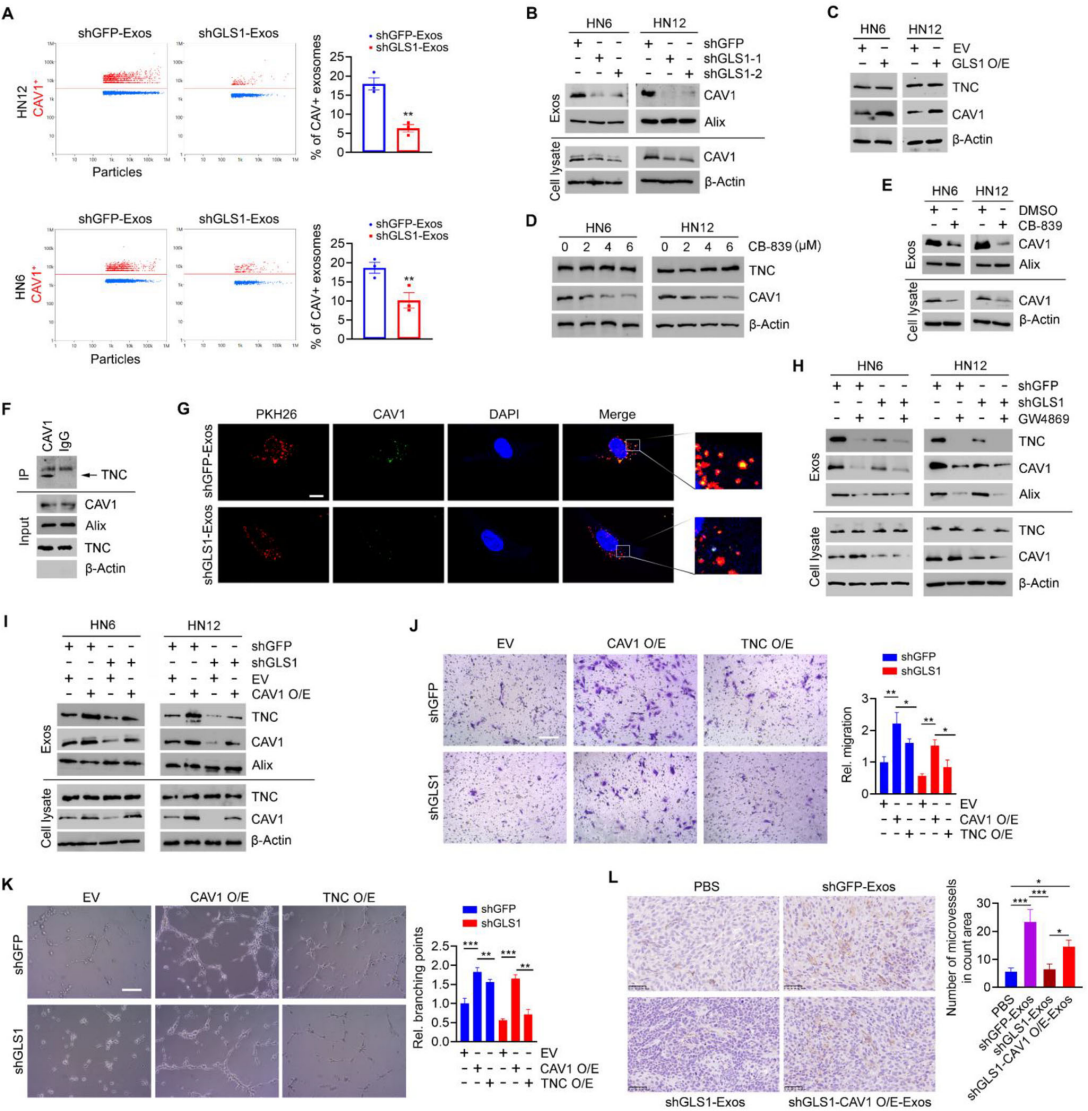
Exosomal encapsulation of TNC is regulated by GLS1 through a CAV1‐dependent process. (A) Percent of CAV1^+^ exosomes in total exosomes derived from *GLS1* knockdown and control HN6 or HN12 cells. Exosomes were stained with anti‐CAV1 antibody followed by an Alexa Fluor 488–conjugated secondary antibody and analyzed by exosome‐flow cytometry. Representative images and quantitative data (n = 3) are shown in the left and right panels, respectively. (B) Effect of *GLS1* knockdown on CAV1 protein levels in HN6 and HN12 cells and their derived exosomes determined by Western blot. (C) Effect of *GLS1* overexpression (O/E) on CAV1 and TNC protein levels in HN6 and HN12 cells determined by Western blot. (D) Effect of varying doses of CB‐839 on CAV1 and TNC protein levels in HN6 and HN12 cells determined by Western blot. (E) Effect of CB‐839 on CAV1 protein levels in HN6 and HN12 cells and their derived exosomes determined by Western blot. (F) IP analysis of the interaction between TNC and CAV1 in exosomes derived from *CAV1*‐overexpressing HN12 cells. Exosomes were enriched and immunoprecipitated with anti‐CAV1 antibody, followed by Western blot analysis using anti‐TNC antibody. Preimmune IgG served as a control. (G) Confocal microscopy images showing internalization of exosomal CAV1 by HUVECs following a 24‐h co‐culture with exosomes derived from *GLS1* knockdown and control HN12 cells. Exosomal CAV1 was stained with anti‐CAV1 antibody (green), exosome membranes were labeled with PKH26 (red), and nuclei were counterstained with DAPI (blue). Scale bar: 20 µm. (H) Effect of GW4869 on CAV1 and TNC protein levels in *GLS1* knockdown and control HN6 or HN12 cells and their derived exosomes determined by Western blot. (I) Effect of *CAV1* overexpression on TNC protein levels in *GLS1* knockdown and control HN6 or HN12 cells and their derived exosomes determined by Western blot. (J) HUVEC migration following a 24‐h transwell incubation with exosomes from *GLS1* knockdown or control HN12 cells with or without CAV1 or TNC overexpression (O/E). Scale bar: 50 µm. (K) HUVEC tube formation following a 24‐h co‐culture with exosomes from *GLS1* knockdown or control HN12 cells with or without CAV1 or TNC O/E. Scale bar: 50 µm. In (J,K), representative images and quantitative data (n = 3) are shown in the left and right panels, respectively. (L) IHC of tumor tissues derived from *GLS1* knockdown HN12 xenografts treated with PBS or exosomes isolated from *GLS1* knockdown (shGLS1‐Exos) or control (shGFP‐Exos) HN12 cells, or *GLS1* knockdown HN12 cells ectopically overexpressing CAV1 (shGLS1‐CAV1 O/E‐Exos), using anti‐CD31 antibody. Quantitative data (n = 5 mice/group) are presented in the right panel. Exosomes were used at a concentration of 100 µg/ml in in vitro study. ^*^ *p* < 0.05; ^**^ *p* < 0.01; ^***^ *p* < 0.001.

CAV1 is a well‐established component of exosomes and has been reported as a critical regulator of exosome cargo sorting, particularly for subsets of ECM proteins [19]. To investigate whether GLS1 regulates exosomal CAV1, we performed exosome flow cytometry and observed a significant reduction in the number of CAV1‐positive exosomes in *GLS1* knockdown HN12 and HN6 cells compared with corresponding control cells (Figure 3A). Western blot analysis further revealed that CAV1 protein was markedly decreased both intracellularly and in exosomes from *GLS1* knockdown cells (Figure 3B). In contrast, overexpression of *GLS1* in HN12 and HN6 cells led to increased CAV1 levels, without affecting TNC expression (Figure 3C). Moreover, CB‐839 treatment resulted in a dose‐dependent suppression of CAV1 protein in both HN12 and HN6 cells (Figure 3D), which was mirrored by reduced CAV1 abundance in exosomes derived from CB‐839‐treated cells (Figure 3E). These findings suggest that the reduction of exosomal CAV1 upon GLS1 inhibition is likely a consequence of decreased intracellular CAV1 levels, rather than a defect in exosome biogenesis per se.

Next, we investigated whether CAV1 was assembled with TNC in tumor‐derived exosomes. Exosomal immunoprecipitation (IP) demonstrated a clear interaction between TNC and CAV1 in exosomes isolated from HN12 cells (Figure 3F). Consistent with our observations for TNC‐positive exosomes (Figure 2D), IF analysis revealed a significant reduction in CAV1‐positive exosomes from *GLS1* knockdown HN12 cells in HUVECs compared with those from control cells (Figure 3G), indicating that GLS1 is critical for the efficient transfer of CAV1‐TNC‐containing exosomes to endothelial cells.

To determine whether inhibition of exosome secretion affects the exosomal protein levels of CAV1 and TNC, HN12 and HN6 cells were treated with GW4869. Both CAV1 and TNC levels were markedly reduced in exosomes isolated from either *GLS1* knockdown or control HNSCC cells following GW4869 treatment (Figure 3H). Interestingly, overexpression of *CAV1* in *GLS1* knockdown HNSCC cells restored the levels of TNC in exosomes that was impaired by *GLS1* depletion, without altering intracellular TNC protein expression (Figure 3I). Functionally, exosomes derived from *GLS1* knockdown HN12 cells with *CAV1* overexpression rescued HUVEC migratory capacity and tube formation (Figure 3J,K). In contrast, overexpression of *TNC* alone in *GLS1* knockdown cells failed to restore exosomal TNC levels (Figure 2F) or HUVEC angiogenic function (Figure 3J,K), suggesting that CAV1 is required for efficient TNC incorporation into exosomes and for mediating its downstream pro‐angiogenic effects downstream of GLS1 signaling.

To in vivo evaluate whether GLS1‐CAV1‐dependent exosomal signaling is essential for the promotion of tumor angiogenesis, NSG mice bearing *GLS1* knockdown HN12 xenografts received intratumoral injection of exosomes isolated from *GLS1* knockdown or control HN12 cells, or *GLS1* knockdown HN12 cells ectopically overexpressing *CAV1*. Exosomes derived from control cells or *GLS1* knockdown cells with *CAV1* overexpression significantly restored tumor angiogenesis compared with the PBS‐treated control group, as demonstrated by increased microvessel density based on CD31 immunostaining (Figure 3L). In contrast, exosomes from *GLS1* knockdown cells failed to rescue angiogenesis (Figure 3L). These results provide direct evidence that GLS1‐dependent exosomal signaling, mediated through CAV1, is required to promote tumor angiogenesis.

### 2.4 GLS1 Promotes CAV1 Protein Stability via Ubiquitin Specific Peptidase 1 (USP1)‐Mediated Deubiquitination

**FIGURE 4:**
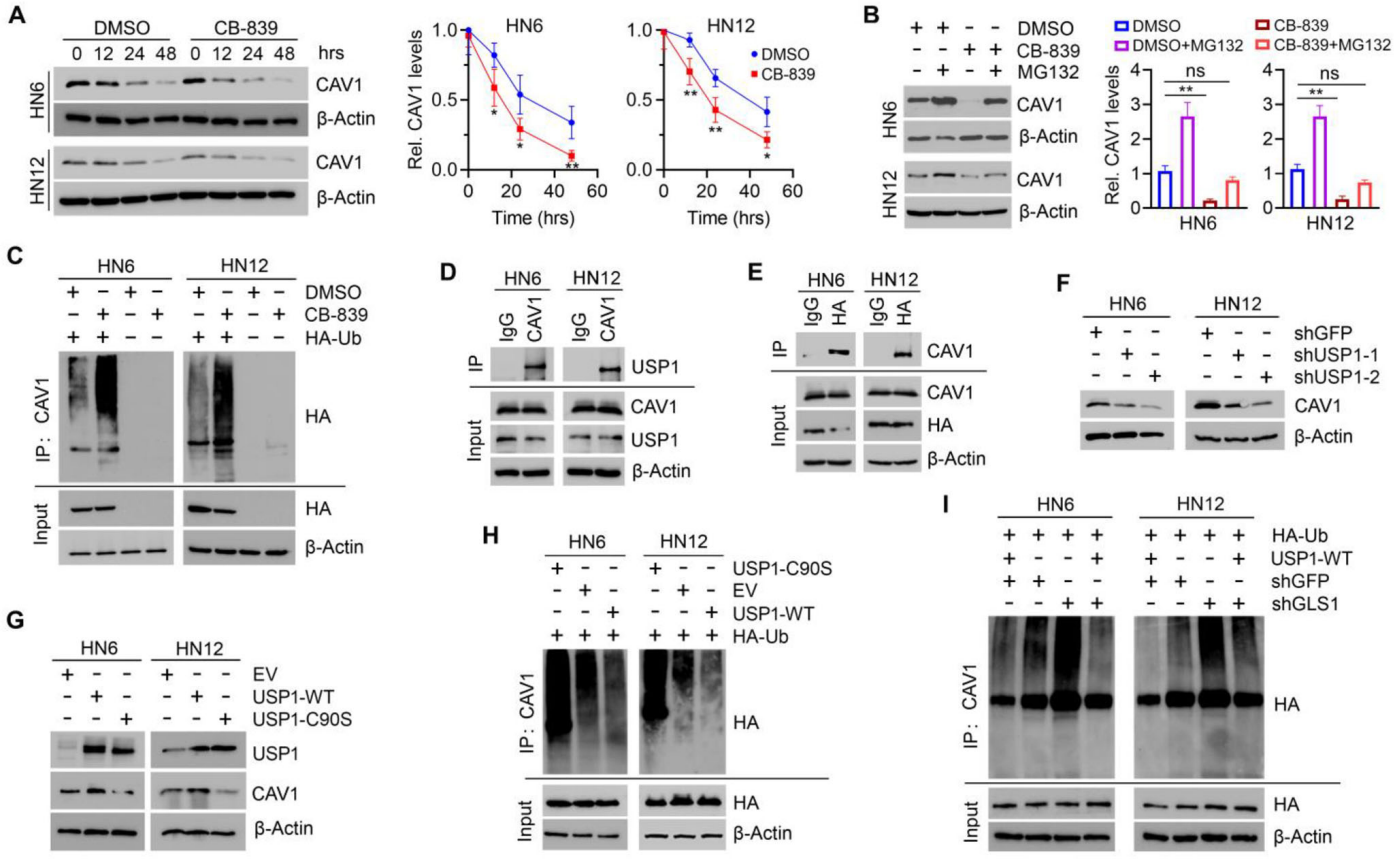
GLS1 stabilizes CAV1 protein by promoting USP1‐mediated deubiquitination. (A) Effect of CB‐839 on the half‐life of CAV1 protein in HN6 and HN12 cells determined by CHX chase assays. Cells were treated with 50 µg/mL CHX for the indicated durations in the presence or absence of 2 µm CB‐839. Representative result and quantitative data (n = 3) are shown in the left and right panels, respectively. (B) Effect of MG132 on CB‐839‐induced reduction of CAV1 protein in HN6 and HN12 cells. Cells were pretreated with 10 µm of the proteasome inhibitor MG132 for 4 h, followed by treatment with 2 µm CB‐839 for 24 h. Quantitative data from three independent experiments are presented in the right panel. (C) Effect of CB‐839 on CAV1 protein ubiquitination in HN6 and HN12 cells. HA‐Ub–transfected cells were pretreated with 10 µm MG132 for 4 h, followed by treatment with 2 µm CB‐839 for 24 h. Cell lysates were immunoprecipitated with anti‐CAV1 antibody and analyzed by HA immunoblotting. (D,E) Interaction between USP1 and CAV1 in HN6 and HN12 cells expressing HA‐USP1. Pre‐immune IgG was used as a negative control. In (D), cell lysates were collected for IP with anti‐CAV1 antibody, followed by USP1 immunoblotting. In (E), cell lysates were collected for IP with anti‐HA antibody, followed by CAV1 immunoblotting. (F) Effect of *USP1* knockdown on CAV1 protein levels in HN6 and HN12 cells. (G) Effect of *USP1* overexpression (WT) and *USP1* enzymatic dead mutant (C90S) on CAV1 protein levels in HN6 and HN12 cells. (H) Effect of *USP1* overexpression (WT) and *USP1* enzymatic dead mutant (C90S) on ubiquitination of CAV1 protein in HN6 and HN12 cells. (I) Effect of restoring *USP1* expression on *GLS1* knockdown‐mediated CAV1 protein ubiquitination in HN6 and HN12 cells. In (H) and (I), the gene‐modified cells were pre‐treated with 10 µM MG132 for 4 h before cell lysates were immunoprecipitated with anti‐CAV1 antibody. ns; not significant; ^*^ *p* < 0.01; **p < 0.01; ***p < 0.001.

Next, we sought to elucidate the mechanism by which *GLS1* loss downregulates CAV1 in HNSCC cells. Our previous RNA‐seq data (GSE240956) revealed that *GLS1* knockdown did not alter *CAV1* mRNA expression in HNSCC cells, implying potential post‐translational regulation of CAV1 by GLS1. To investigate this possibility, HN6 and HN12 cells were treated with cycloheximide (CHX) to inhibit *de novo* protein synthesis, with or without CB‐839. Treatment with CB‐839 significantly reduced the half‐life of CAV1 protein compared with control cells (Figure 4A), indicating that GLS1 inhibition reduces the stability of CAV1 protein. Furthermore, treatment with the proteasome inhibitor MG132 restored CAV1 protein levels in CB‐839‐treated HNSCC cells (Figure 4B), suggesting that GLS1 inhibition promotes CAV1 degradation through the ubiquitin‐proteasome system. To directly evaluate CAV1 ubiquitination, HN6 and HN12 cells overexpressing HA‐tagged *ubiquitin* (HA‐Ub) were pretreated with MG132 and subsequently exposed to CB‐839. IP with anti‐CAV1 antibody followed by HA immunoblotting showed elevated levels of ubiquitinated CAV1 in CB‐839‐treated cells (Figure 4C).

Our previous study demonstrated that *GLS1* knockdown in HNSCC cells led to significant alterations in the expression of multiple ubiquitin‐related genes [9]. Among them, *USP1* was identified as the most downregulated gene encoding a deubiquitinating enzyme. Given that USP1 is known to regulate protein stability by removing ubiquitin moieties from specific substrates, it may contribute to the maintenance of CAV1 stability. Co‐IP analysis revealed that CAV1 was bound to endogenous or exogenously expressed USP1 in HN12 and HN6 cells (Figure 4D,E). CAV1 was also found to associate with USP1 in 293T cells, indicating that this protein‐protein interaction is not restricted to a specific cancer cell type (Figure S8). Moreover, *USP1* knockdown in HNSCC cells led to a pronounced decrease in CAV1 protein levels (Figure 4F), whereas *USP1* overexpression increased CAV1 abundance (Figure 4G). In contrast, overexpression of the catalytically inactive *USP1* mutant (C90S) [20] resulted in reduced CAV1 levels in HNSCC cells (Figure 4G). Consistently, enhanced CAV1 ubiquitination was observed in cells expressing the *USP1* C90S mutant, while wild‐type *USP1* expression attenuated CAV1 ubiquitination (Figure 4H), supporting the notion that USP1 stabilizes CAV1 by deubiquitinating it and preventing its proteasomal degradation. Most importantly, restoration of *USP1* expression in *GLS1* knockdown HNSCC cells decreased the proportion of ubiquitinated CAV1 (Figure 4I). These findings indicate that *GLS1* loss downregulates *USP1* expression, leading to enhanced CAV1 ubiquitination and protein degradation in HNSCC cells.

### 2.5 Exosomes Derived From *GLS1‐*Deficient HNSCC Cells Attenuate Angiogenesis by Suppressing FAK‐SRC Signaling in Endothelial Cells

**FIGURE 5:**
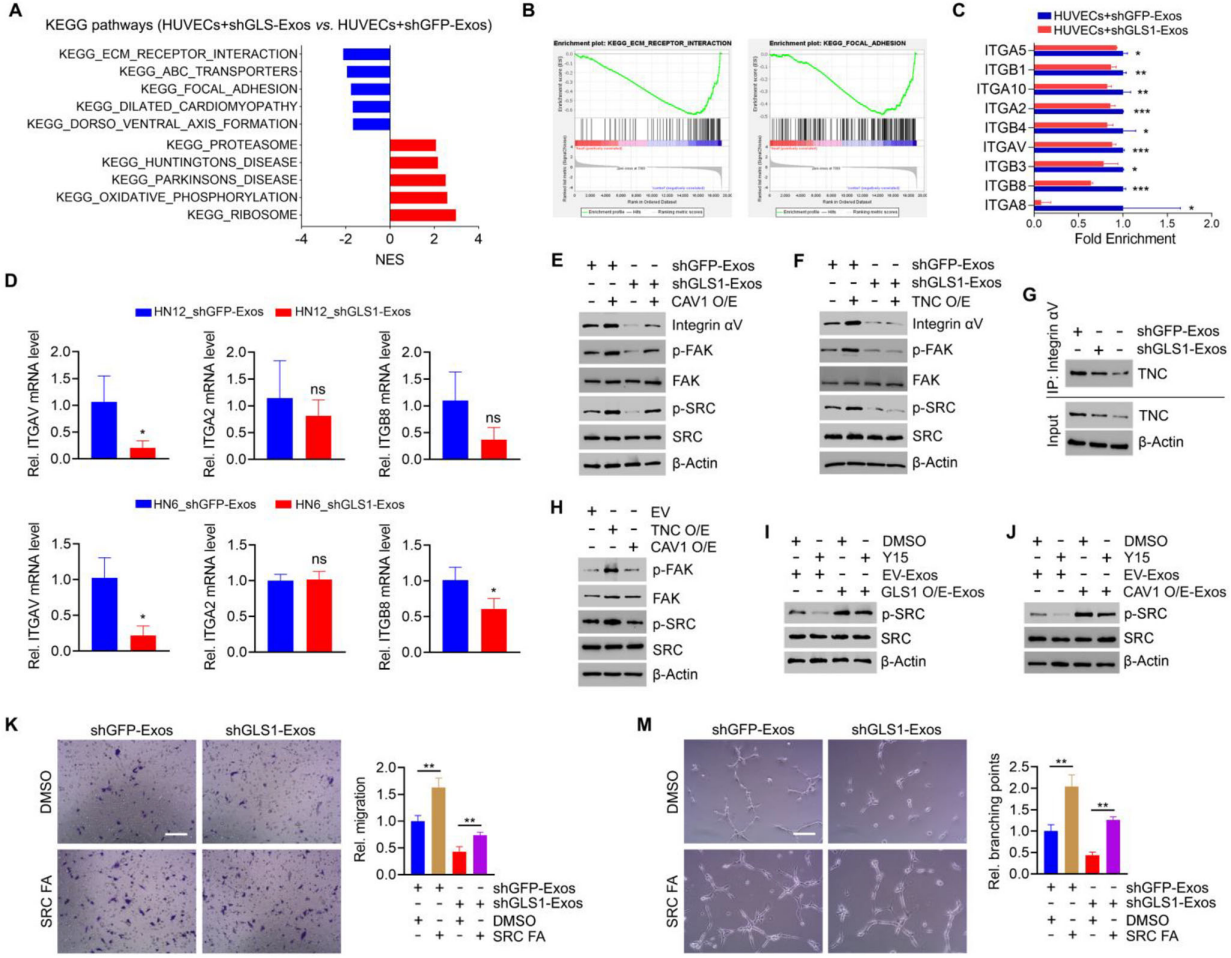
Exosomes derived from *GLS1*‐deficient HNSCC cells block angiogenesis via FAK‐SRC signaling inhibition in endothelial cells. (A) RNA‐seq‐based KEGG pathway enrichment analysis of differentially expressed genes (DEGs) in HUVECs following a 24‐h co‐culture with exosomes derived from *GLS1* knockdown (shGLS1‐Exos) and control (shGFP‐Exos) HN12 cells. Top five most upregulated and downregulated KEGG pathways are presented. (B) Downregulated enrichment plots for the KEGG_ECM_RECEPTOR_INTERACTION and KEGG_FOCAL_ADHESION gene sets in HUVECs co‐cultured with exosomes from GLS1 knockdown HN12 cells compared to control exosomes. (C) Fold change of the nine most downregulated integrin genes in HUVECs based on RNA‐seq data. (D) RT‐qPCR analysis of *ITGAV*, *ITGA2*, and *ITGB8* expression in HUVECs after a 24‐h co‐culture with exosomes from *GLS1* knockdown or control HN12 or HN6 cells. (E) Changes in FAK and SRC phosphorylation in HUVECs following a 24‐h co‐culture with exosomes from *GLS1* knockdown or control HN12 or HN6 cells with or without *CAV1* overexpression (CAV1 O/E). (F) Changes in FAK and SRC phosphorylation in HUVECs following a 24‐h co‐culture with exosomes from *GLS1* knockdown or control HN12 or HN6 cells with or without *TNC* overexpression (TNC O/E). (G) IP analysis of the interaction between TNC and integrin αV in HUVECs co‐cultured with exosomes from *GLS1* knockdown or control HN12 cells. HUVEC cell lysates were immunoprecipitated with anti‐integrin αV antibody, followed by Western blot analysis using anti‐TNC antibody. HUVECs cultured alone served as a control. (H) Changes in FAK and SRC phosphorylation in HUVECs with or without TNC or CAV1 overexpression. (I) Effect of 10 µM Y15 in SRC phosphorylation in HUVECs following a 24‐h co‐culture with exosomes from *GLS1* overexpressing (GLS1 O/E‐Exos) or control (EV‐Exos) HN12 cells. (J) Effect of 10 µM Y15 in SRC phosphorylation in HUVECs following a 24‐h co‐culture with exosomes from *CAV1* overexpressing (CAV1 O/E‐Exos) or control (EV‐Exos) HN12 cells. (K) Effect of 20 µM SRC Family Activator (FA) on HUVEC migration following a 24‐h transwell incubation with exosomes from *GLS1* knockdown or control HN12 cells. (L) Effect of 20 µM SRC FA on HUVEC tube formation following a 24‐h co‐culture with exosomes from *GLS1* knockdown or control HN12 cells. (K,L) Representative images and quantitative data (n = 3) are shown in the left and right panels, respectively. Exosome concentration used in this study is 100 µg/ml. Scale bar: 50 µm. ^*^ *p* < 0.05; ^**^ *p* < 0.01; ^***^ *p* < 0.001.

To gain an unbiased understanding of how GLS1‐driven exosomes influence endothelial cell biology, we performed RNA‐seq analysis using RNA extracted from HUVECs co‐cultured with exosomes derived from *GLS1* knockdown or control HN12 cells. A total of 418 differentially expressed genes (DEGs) were identified based on the criteria of |log_2_FC|≥1 and *p* < 0.05 (Figure S9). KEGG pathway enrichment analysis revealed that the *KEGG_ECM_RECEPTOR_INTERACTION* and *KEGG_FOCAL_ADHESION* gene sets were among the top five downregulated pathways in *GLS1* knockdown HN12 cells (Figure 5A,B). Given that integrins play a central role in mediating both pathways, we conducted a focused analysis of integrin family members using RNA‐seq data. This analysis identified *ITGA2*, *ITGA8*, and *ITGAV* as among the most significantly downregulated integrin genes in HUVECs exposed to exosomes from *GLS1* knockdown HN12 cells (Figure 5C). Subsequent RT‐qPCR validation revealed that only *ITGAV* expression was consistently reduced in HUVECs co‐cultured with exosomes from *GLS1* knockdown HNSCC cells (Figure 5D), suggesting that integrin αV (encoded by *ITGAV*) may serve as a mediator of GLS1‐dependent exosomal signaling in regulating endothelial cell function.

We next examined the activation status of FAK and SRC, two central kinases downstream of integrin signaling. HUVECs co‐cultured with exosomes from *GLS1* knockdown HN12 cells exhibited a marked reduction in FAK and SRC phosphorylation (Figure 5E). This signaling deficit in HUVECs was rescued by restoring exosomal CAV1 levels, as co‐culture with exosomes from *GLS1* knockdown cells overexpressing *CAV1* reinstated robust FAK and SRC phosphorylation (Figure 5E). However, FAK‐SRC signaling activation was not restored when HUVECs were co‐cultured with exosomes from *GLS1* knockdown cells overexpressing *TNC* (Figure 5F), suggesting that CAV1 is required for exosomal TNC‐mediated activation of the FAK‐SRC pathway in endothelial cells. IP analysis revealed an interaction between TNC and integrin αV in HUVECs (Figure S10). Notably, an increased amount of TNC protein was found bound to integrin αV in HUVECs co‐cultured with exosomes derived from HN12 control cells, whereas TNC binding to integrin αV was dramatically reduced in HUVECs co‐cultured with exosomes from *GLS1* knockdown HN12 cells (Figure 5G).

Moreover, overexpression of *TNC*, but not *CAV1*, in HUVECs enhanced FAK and SRC phosphorylation (Figure 5H), supporting its role in activating integrin‐mediated signaling. When HUVECs were treated with the FAK inhibitor Y15 or the SRC inhibitor AZD0530, either of them effectively suppressed SRC phosphorylation and markedly reduced HUVEC migratory capacity (Figure S11). Furthermore, HUVECs were treated with Y15 while co‐cultured with exosomes from *GLS1*‐ or *CAV1*‐overexpressing HN12 cells or their corresponding controls. Y15 effectively abolished SRC phosphorylation regardless of the exosome source (Figure 5I,J), demonstrating that the activation of FAK‐SRC signaling is essential for mediating tumor angiogenesis driven by tumor‐intrinsic GLS1. In addition, treatment with a SRC family activator (SRC FA) rescued the impaired migration and tube formation of HUVECs caused by co‐culture with exosomes from *GLS1* knockdown HN12 cells (Figure 5K–M). Collectively, these findings indicate that TNC, packaged into tumor‐derived exosomes through CAV1 under GLS1 regulation, interacts with integrin αV on endothelial cells to activate FAK‐SRC signaling, which is essential for promoting endothelial cell migration and angiogenic tube formation.

### 2.6 Targeting GLS1 in HNSCC PDOs Impairs Angiogenic Potential Through Tumor‐Derived Exosomes

**FIGURE 6:**
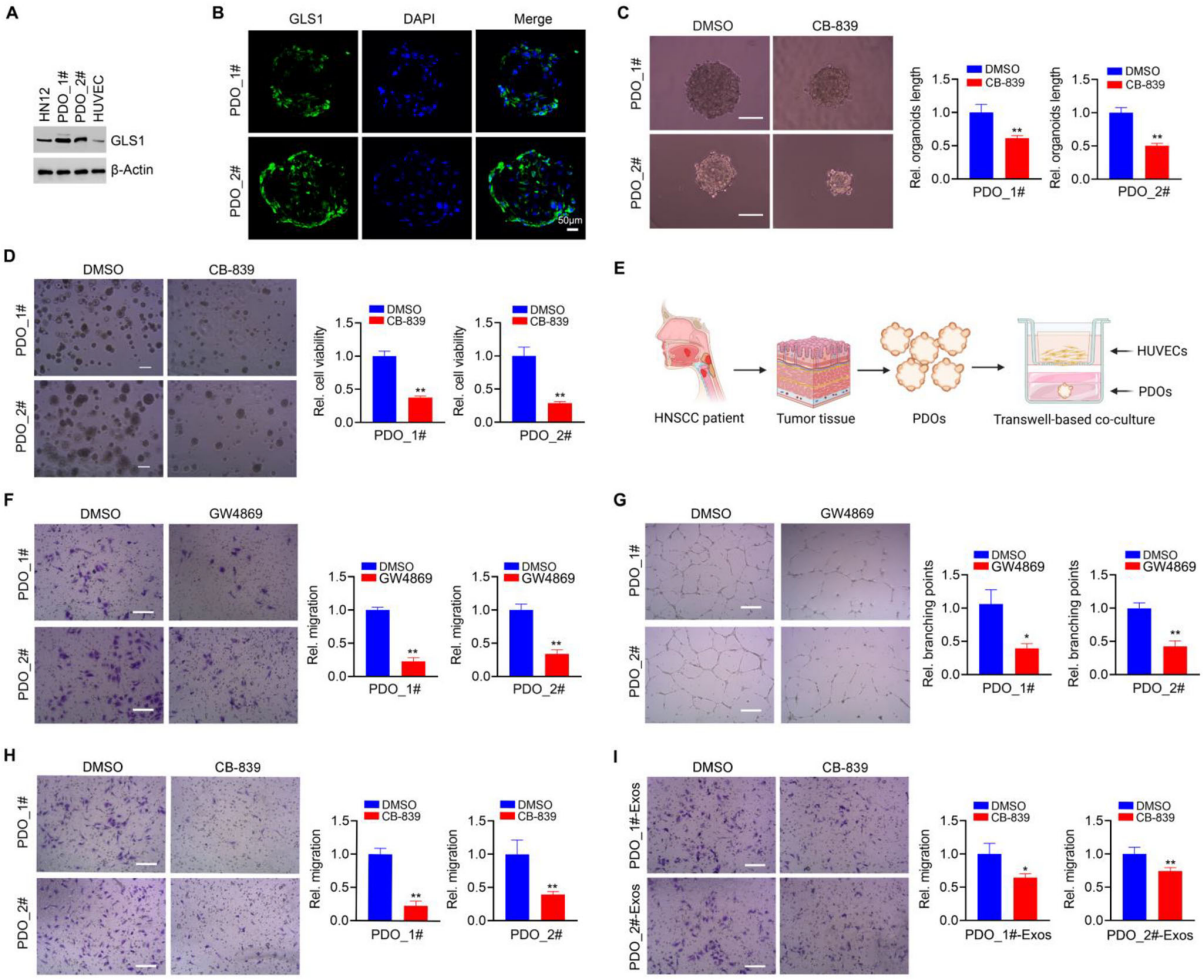
Targeting GLS1 in HNSCC PDOs suppresses angiogenic potential through exosomes. (A) GLS1 protein levels in HN12 cells, HNSCC PDOs (PDO_1# and PDO_2#), and HUVECs determined by Western blot. (B) Confocal microscopy images showing GLS1 levels in two PDOs. PDOs were stained with anti‐GLS1 antibody followed by an Alexa Fluor 488‐conjugated secondary antibody (green); nuclei were counterstained with DAPI (blue). Scale bar: 50 µm. (C, D) Effect of 5 µm CB‐839 on the growth of HNSCC PDOs after 7 days of treatment. (C) Representative images of individual organoids under different treatment conditions (left) with corresponding quantitative analysis of organoid length (right, n = 10). Scale bar: 100 µm. (D) Representative images of pooled organoids treated with 5 µm CB‐839 or DMSO (left) and quantification of cell viability (right, n = 3). Scale bar: 200 µm. (E) Schematic illustration of Transwell‐based co‐culture system of HUVECs with HNSCC PDOs. PDOs generated from patient tumor specimens were seeded in the lower chamber of the Transwell, and HUVECs were seeded in the upper insert for co‐culture. Figure created using Biorender (https://biorender.com). (F) HUVEC migration following a 24‐h transwell incubation with PDOs treated with or without 20 µm GW4869 for 24 h. (G) HUVEC tube formation following a 24‐h culture in the conditioned media collected from PDOs pretreated with or without 20 µm GW4869 for 24 h. (H) HUVEC migration following a 24‐h transwell incubation with PDOs pretreated with or without 5 µm CB‐839 for 7 days. (I) HUVEC migration following a 24‐h transwell incubation with exosomes from PDOs pretreated with or without 5 µm CB‐839 for 7 days. In (F‐I), representative images and quantitative data (n = 3) are shown in the left and right panels, respectively. Exosomes were used at a concentration of 100 µg/ml in this study. Scale bar: 50 µm. ^*^ *p* < 0.05; ^**^ *p* < 0.01.

Importantly, *GLS1* was abundant in both HNSCC PDOs (PDO_1# and PDO_2#) used in this study (Figure 6A,B), with expression level much higher than that detected in HUVECs (Figure 6A). To evaluate the therapeutic potential of targeting GLS1 in clinically relevant models, these two PDOs were treated with CB‐839, which resulted in a marked reduction in organoid size and cell viability compared to control groups (Figure 6C,D). To better investigate the role of PDO‐driven exosomes in modulating endothelial cell function, we established a transwell‐based PDO‐HUVEC co‐culture system (Figure 6E). In this system, pharmacological inhibition of exosome biogenesis in PDOs using GW4869 impaired HUVEC migration and tube formation (Figure 6F,G). Furthermore, HUVECs co‐cultured with CB‐839‐treated PDOs exhibited significantly reduced migratory capacity compared to those co‐cultured with control groups (Figure 6H), indicating that GLS1 activity in PDOs contributes to endothelial cell motility. To assess whether this effect was mediated by GLS1‐regulated exosomes, we isolated exosomes from PDOs treated with either CB‐839 or DMSO. HUVECs co‐cultured with exosomes from CB‐839‐treated PDOs exhibited markedly reduced migratory capacity compared with those exposed to control exosomes (Figure 6I). These results suggest GLS1 inhibition as a potential strategy to disrupt tumor‐endothelial crosstalk.

## 3 Discussion

**FIGURE 7:**
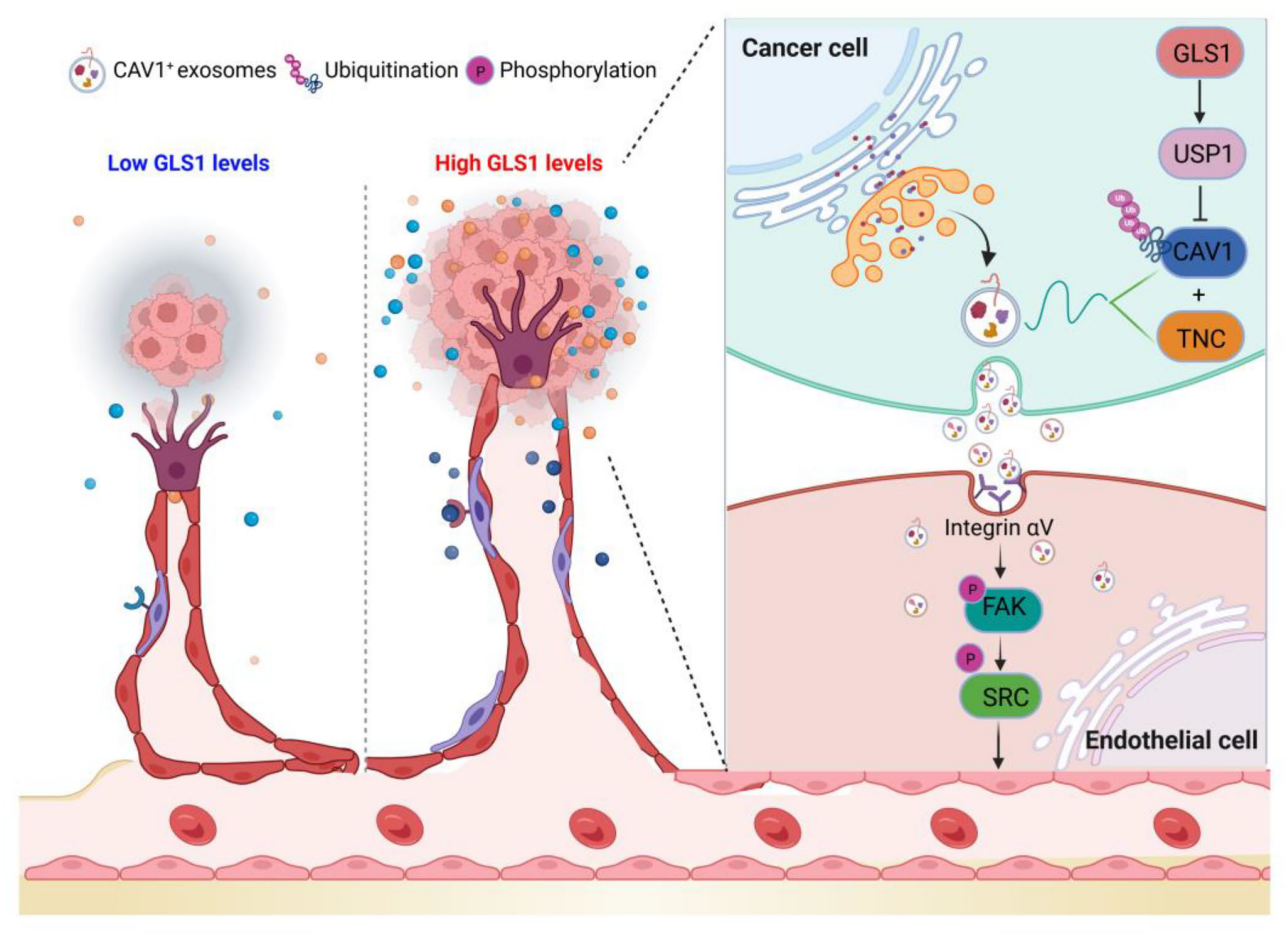
Schematic of GLS1‐driven exosome signaling that facilitates communication between tumor cells and the vascular microenvironment to enhance angiogenesis. *GLS1* upregulation in HNSCC cells stabilizes CAV1 protein by promoting USP1‐mediated deubiquitination. The resulting accumulation of CAV1 facilitates the assembly of CAV1‐TNC complexes into tumor‐derived exosomes. These CAV1‐TNC‐enriched exosomes exhibit heightened binding affinity to integrin αV on endothelial cells, thereby activating the FAK‐SRC signaling cascade and promoting endothelial cell activation and angiogenesis. Figure created using Biorender (https://biorender.com).

HNSCC is characterized by a highly vascularized TME driven by both tumor‐intrinsic drivers and microenvironmental signals [21, 22]. Dysregulated pro‐angiogenic signaling, including VEGF‐ and hypoxia‐driven pathways, contributes to aggressive tumor behavior and therapeutic resistance [23, 24]. Targeting angiogenesis has demonstrated clinical relevance in HNSCC, with emerging evidence supporting the use of VEGF tyrosine kinase inhibitors. However, the modest efficacy of current anti‐angiogenic agents highlights the complexity of angiogenic regulation in HNSCC and the need to identify novel mediators. In this study, we demonstrate that upregulation of *GLS1* in HNSCC cells exerts a previously unrecognized regulatory function beyond its canonical metabolic role. Specifically, GLS1 enhances the stability of CAV1 by promoting USP1‐mediated deubiquitination, leading to the accumulation of CAV1 protein in HNSCC cells. The increased CAV1 levels facilitate the assembly of CAV1‐TNC complexes that are selectively packaged into tumor‐derived exosomes (Figure 7). These TNC‐enriched exosomes trigger activation of FAK‐SRC signaling at least partially via integrin αV in endothelia cells, promoting their migration and angiogenic remodeling within the TME (Figure 7). To provide strong evidence that GLS1‐CAV1‐associated exosome signaling drives tumor angiogenesis, we employed an in vivo exosome add‐back approach. Although such experiments are inherently challenging due to limitations in exosome delivery, stability, and biodistribution, the intramucosal location of our tumor model conferred a significant experimental advantage. Intratumoral delivery of exosomes allowed us to bypass systemic barriers, reduce off‐target distribution and degradation, and achieve localized, biologically relevant exposure. This strategy not only reinforces the causal role of GLS1‐CAV1 exosomal signaling in angiogenesis but also provides a practical framework for evaluating exosome‐based therapies in accessible solid tumors.

Metabolic activities of cancer cells profoundly remodel the TME, not only by altering nutrient and oxygen gradients but also by secreting EVs such as exosomes that mediate intercellular communication and microenvironmental adaptation [25, 26, 27]. Because GLS1 is a key enzyme regulating glutaminolysis, we investigated whether glutamine metabolism influences CAV1‐TNC signaling and its impact on endothelial activation. Under glutamine‐deprived conditions, both CAV1 protein levels in HNSCC cells and TNC abundance in their derived exosomes were markedly reduced (Figure S12A,B), indicating that active glutaminolysis is required to sustain CAV1‐TNC signaling in tumor‐derived exosomes. Consistent with this, HUVECs showed reduced uptake of exosomes derived from glutamine‐deprived tumor cells compared with those from glutamine‐replete conditions (Figure S12C), suggesting that metabolic stress impairs exosome integrity or receptor‐mediated endocytosis. Functionally, exosomes from glutamine‐deprived HNSCC cells significantly reduced HUVEC migration and tube formation relative to control exosomes (Figure S12D,E). These novel findings demonstrate that glutamine availability and GLS1‐mediated glutaminolysis are essential for maintaining CAV1‐TNC‐dependent exosomal signaling, which in turn promotes endothelial uptake, motility, and angiogenic remodeling within the TME.

CAV1 plays a crucial role in shaping the molecular composition of exosomes by regulating the selective loading of specific cargo. Previous studies have established CAV1 as a key organizer of exosomal biogenesis, where it influences membrane dynamics and cargo sorting within multivesicular bodies (MVBs) [19, 28, 29]. Particularly, CAV1 has been shown to facilitate the selective incorporation of TNC into exosomes through cholesterol‐dependent membrane remodeling, a process that governs the sorting of TNC and other ECM components into distinct exosomal subpopulations [19]. Through this mechanism, CAV1 determines both the composition and functional capacity of tumor‐derived exosomes, thereby modulating signaling to recipient cells, including endothelial cells, in the TME. Consistent with these reports, our findings support a model in which CAV1 is required for efficient exosomal TNC packaging in HNSCC cells. However, the present study does not distinguish whether CAV1 mediates this process through direct cargo‐sorting interactions or indirectly by regulating membrane organization and exosome biogenesis. Dissecting these mechanisms represents an important direction for future investigation.

The present study identifies, for the first time, GLS1 as a key regulator of CAV1 protein stability in HNSCC cells. Although the precise upstream mechanisms by which *GLS1* loss leads to reduced *USP1* expression remain to be fully defined, our findings establish a clear functional axis in which GLS1 activity promotes USP1‐dependent stabilization of CAV1 and subsequent pro‐angiogenic signaling. We acknowledge that the regulatory pathways connecting GLS1 to *USP1* gene expression, potentially involving metabolic stress responses, redox signaling, or transcriptional regulation, are not fully delineated in the current study. To address this limitation, ongoing and future investigations are focused on elucidating the metabolic and transcriptional mechanisms through which GLS1 regulates *USP1* expression in HNSCC cells.

TNC is highly overexpressed across a range of malignant solid tumors, including breast cancer, lung cancer, and HNSCC, as well as in certain hematological malignancies, while its expression is minimal or absent in most normal tissues [30, 31]. TNC is particularly enriched in the tumor ECM and hyperplastic blood vessels, where it serves as a key regulator of angiogenesis [32, 33]. Previous studies have demonstrated that TNC induces epithelial‐mesenchymal transition (EMT)‐like changes in breast cancer cells through its interaction with integrins αvβ6 and αvβ1. The cooperative binding of these integrins to TNC activates focal adhesion signaling and FAK phosphorylation, establishing TNC as a key extracellular regulator of EMT in the TME [34]. In the present study, we show that TNC‐enriched tumor‐derived exosomes interact with integrin αV on endothelial cells to activate FAK‐SRC signaling, positioning exosomal TNC as a critical “angiogenic switch” in HNSCC. Importantly, this mechanism appears to function independently of canonical VEGF signaling, providing a potential explanation for the limited efficacy and emergence of resistance observed with anti‐VEGF therapies in solid tumors.

While our study provides novel insights into GLS1‐driven exosome‐mediated angiogenesis, several limitations should be acknowledged. First, most mechanistic experiments were conducted using HNSCC cell‐HUVEC and PDO‐HUVEC co‐culture models. Although these systems capture key aspects of tumor‐endothelial interactions, they do not fully recapitulate the complexity of the TME, such as contributions from immune cells and stromal heterogeneity [35, 36, 37]. Second, our investigation focused primarily on HNSCC models. Given that *GLS1* is broadly expressed across multiple cancer types, further studies are warranted to determine whether this exosome‐mediated angiogenic mechanism is conserved in other tumor contexts. Finally, while our findings suggest that GLS1‐driven angiogenic activity is independent of VEGF and that blocking GLS1 may complement existing anti‐angiogenic therapies, we did not perform preclinical therapeutic studies to assess efficacy, toxicity, or potential combinatorial strategies, such as combining CB‐839 with anti‐VEGF agents.

In conclusion, this study uncovers a previously unrecognized role of GLS1 in promoting angiogenesis in HNSCC through the regulation of exosome‐mediated signaling. Targeting GLS1 could therefore disrupt tumor vascularization and growth, supporting the development of effective therapeutic strategies with potential clinical benefit in HNSCC and other solid tumors.

## 4 Methods

### 4.1 Cell Culture

HN6 and HN12 cells were generously provided by Dr. Andrew Yeudall, and MOC2 cells were obtained from Kerafast. These cell lines were cultured in DMEM/F12 medium (Gibco, Cat# 11320033) supplemented with 5% FBS (Hyclone, Cat# 16777‐002) and 1% penicillin‐streptomycin (Gibco, Cat# 15240062). 293T cells and HUVECs were purchased from ATCC. 293T cells were cultured in DMEM, whereas HUVECs were maintained in EGM‐2 Endothelial Cell Growth Medium‐2 BulletKit (Lonza, Cat# CC‐CC‐3162). Cells were used for experiments before passage 10 and were routinely screened for mycoplasma contamination by MycoAlert Mycoplasma Detection Kit (ATCC, Cat# 30–1012K).

### 4.2 Tumor Dissociation and PDO Culture

All clinical specimens were obtained with written informed consent from the patients, in accordance with an Emory University Institutional Review Board (IRB)‐approved protocol. Tumor tissues from HNSCC patients were excised and immediately placed on ice in DMEM/F12 medium. After three washes with ice‐cold DPBS containing 100 µg/ml Primocin (InvivoGen, Cat# ant‐pm‐05), tissues were minced into ∼2 mm fragments and digested in DMEM/F12 supplemented with 5 mg/ml collagenase II and 10 µM Y‐27632 for 1 h at 37°C. The digested samples were centrifuged, and the supernatant was removed. Pellets were further dissociated in 1 ml TrypLE (Thermo Fisher, Cat# 12‐605‐010) containing 10 µm Y‐27632 for 10 min at 37°C. The resulting single‐cell suspension was filtered through a 70 µm strainer, resuspended, and seeded at 1 × 10^5^ cells in 50 µl of Matrigel (Corning, Cat# 356234). After polymerization at 37°C for 30 min, cells were overlaid with supplemented OncoPro medium (Thermo Fisher, Cat# A5701201) containing 10 µm Y‐27632. Medium was replaced every other day. PDOs were harvested for analysis once they reached ∼200 µm in diameter, as measured with ImageJ. Cell viability of PDOs was determined using the CellTiter‐Glo Luminescent Cell Viability Assay (Promega, Cat# G7571).

### 4.3 Reagents, Antibodies, Constructs, and Standard Assays

CHX and MG132 were obtained from Sigma–Aldrich (St Louis, MO). Y15, AZD0530, and GW4869 were obtained from MedchemExpress (Monmouth Junction, NJ). CB‐839 was purchased from Selleckchem (Houston, TX). SRC Family Activator (EPQpYEEIPIYL) was purchased from Santa Cruz Biotechnology (Dallas, TX). pLKO.1‐puro TRC control shRNA targeting the green fluorescent protein gene (shGFP), as well as specific shRNAs targeting human *GLS1* (shGLS1) or *USP1* (shUSP1) genes were obtained from Horizon Discovery (Waterbeach, UK). shRNAs targeting the mouse *Gls* gene (shGls) were purchased from VectorBuider Inc. (Chicago, IL). ViraPower Lentiviral Packaging Mix, containing an optimized mixture of the three packaging plasmids (pLP1, pLP2, and pLP/VSVG), was purchased from Invitrogen (Carlsbad, CA). Human *GLS1* cDNA‐flag was purchased from Sino Biological (China). Human *TNC* cDNA was amplified from a pBS‐HxB.L, which was a gift from Harold Erickson (Addgene plasmid #65414; http://n2t.net/addgene:65414; RRID:Addgene_65414) [38], and then cloned into a pcDNA3.1(+) vector (Invitrogen, Cat# V79020). pAD‐CMV‐Caveolin1‐CMV‐GFP was a gift from Andrew Brooks (Addgene plasmid #83272; http://n2t.net/addgene:83272; RRID:Addgene_83272) [39]. Full‐length HA‐tagged human ubiquitin expression plasmid (HA‐Ub) was provided by Dr. Shi‐Yong Sun at Emory University, and Full‐length HA‐tagged human USP1 expression plasmid (HA‐USP1) was a kind gift from Dr. Jianmin Zhang at Roswell Park Comprehensive Cancer Center. Full‐length human USP1 (USP1‐WT) and its enzymatic dead mutant (USP1‐C90S) plasmids were provided by Dr. Tony T. Huang at New York University [20]. All antibodies and primers used are listed in Tables S2 and S3, respectively. Plasmid transfection and lentiviral infection, RT‐qPCR, Western blot, CHX chase assay, IP, and cellular ubiquitination assays were conducted as we previously described [1, 9].

### 4.4 Exosome Isolation

Exosome‐depleted FBS was prepared by ultracentrifugation at 120 000 × *g* for 18 h at 4°C using a 45Ti rotor (Beckman Coulter), and the resulting supernatant was collected. HNSCC cells were cultured in media supplemented with 10% exosome‐depleted FBS for 48 h. Exosomes were isolated from cell culture supernatants by sequential centrifugation at 4°C. Briefly, culture medium was centrifuged at 300 × *g* for 10 min to remove intact cells, followed by centrifugation at 2000 × *g* for 10 min and 10 000 × *g* for 30 min to eliminate cell debris. The supernatant was passed through a 0.22 µm filter, and exosomes were pelleted by ultracentrifugation at 100 000 × *g* for 120 min (Beckman Type 45 Ti). The pellet was washed with cold PBS and subjected to a second ultracentrifugation under the same conditions. The final exosome pellet was resuspended in 300 µl PBS for downstream analyses, including protein quantification, nanoparticle tracking, or functional assays.

### 4.5 LC‐MS

Exosomes isolated from *GLS1* knockdown or control HN12 cells were resuspended in PBS and subjected to LC‐MS analysis at the Taplin Mass Spectrometry Facility (Harvard Medical School). LC‐MS data were processed using MaxQuant software (version 2.5.2.0; RRID: SCR_014485). Peak lists were generated for comprehensive database searching, and DSPs were identified through filtering on fold change (FC)≥2 and *p* <0.05. The resulting data were visualized on a two‐dimensional plot with retention time on the x‐axis and mass‐to‐charge ratio (m/z) on the y‐axis, providing a clear representation of protein distribution and abundance.

### 4.6 Endothelial Cell Migration Assay

HUVECs were seeded at 2 × 10^4^ cells per 100 µl of serum‐free medium into the upper chamber of Transwell inserts (8.0 µm pore size, Corning, USA). For exosome incubation, exosomes isolated from genetically modified HNSCC cells or from cells/PDOs treated with or without the indicated treatment were diluted in DMEM/F‐12 medium supplemented with 10% exosome‐depleted FBS and added to the lower chamber at a final concentration of 100 µg/ml in 700 µl of medium. For conditioned media incubation, conditioned media collected from genetically modified HNSCC cells or from cells/PDOs treated with or without the indicated treatment was concentrated by centrifugation at 3000 × g for 1 h using 3 kDa MWCO filters and then added to the lower chamber in 700 µl volume. After 24 h of incubation, the medium in the upper chamber was removed, and the inserts were fixed with 4% paraformaldehyde for 20 min at room temperature. Membranes were then stained with 0.5% crystal violet for 30 min, rinsed with distilled water, and the non‐migrated cells on the upper surface were gently removed with a cotton swab. Inserts were air‐dried and examined under a light microscope. Three bright‐field images per membrane were acquired using a 10× objective. Migrated cells were quantified manually by counting stained cells in the images with ImageJ software (NIH). For migration assays in the PDO‐HUVEC co‐culture system, 1 × 10^5^ PDO cells embedded in 50 µl of Matrigel were seeded in the lower chamber. After gel solidification, fresh medium was added, and PDOs were treated with or without 5 µm CB‐839 for 7 days. HUVECs were then seeded in serum‐free medium into the upper chamber of Transwell inserts and co‐cultured for 24 h prior to analysis.

### 4.7 Endothelial Cell Tube Formation Assay

Approximately 250 µl of Matrigel (BD Biosciences) was added to each well of a 24‐well plate and incubated at 37°C for 30 min to allow polymerization. 1 × 10^5^ HUVECs were resuspended in medium, seeded into the Matrigel‐coated wells, and incubated with exosomes derived from different conditions for 24 h. Tube formation was assessed under a light microscope, and both the number and total length of tubular structures were quantified from three randomly selected fields per well using ImageJ software (NIH).

### 4.8 IF

To monitor exosome trafficking, exosomes were labeled with the PKH26 Red Fluorescent Cell Linker Kit (Sigma–Aldrich). HUVECs were incubated with labeled exosomes at concentrations of 50 µg/ml for 24 h. Cells were fixed with 4% paraformaldehyde in PBS and permeabilized with 0.1% Triton X‐100 for 20 min. After blocking, slides were incubated overnight at 4°C with primary antibodies against TNC or CD31, followed by incubation with fluorescence‐conjugated secondary antibodies for 1 h in the dark. In a separate set of experiments designed to detect specific proteins in the exosomes, exosomes were first incubated overnight at 4°C with primary antibodies against TNC or CAV1, followed by incubation with fluorescence‐conjugated secondary antibodies. Subsequently, these exosomes were labeled with PKH26 and purified via ultracentrifugation before being co‐cultured with HUVECs for 24 h. Cells were then fixed, permeabilized, and stained with DAPI. All samples were mounted with Vectashield mounting medium (Vector Laboratories) containing DAPI for nuclear staining and examined using a ZEISS confocal laser scanning microscope (Dublin, CA).

### 4.9 Measurement of Exosome Size Distribution

Exosomes isolated from *GLS1* knockdown or control HN12 cells were diluted in 0.22 µm filtered PBS to reach the optimal concentration recommended by the manufacturer. Polystyrene nanobead mixtures of defined diameters (NanoFCM, China) were used to calibrate side scatter signals and establish the size standard curve. Data were collected with NanoFCM software and converted to particle size distribution.

### 4.10 Flow Cytometric Analysis of CAV1‐Positive Exosomes

Approximately 5 × 10^8^ exosome particles per sample were used. Exosomes were incubated with a primary antibody against CAV1 for 30 min at room temperature in the dark, followed by incubation with a fluorophore‐conjugated secondary antibody (anti‐IgG, Alexa Fluor/PE, Thermo Fisher) for 30 min under the same conditions. Labeled exosomes were analyzed using a NanoAnalyzer (NanoFCM Inc., China). Size‐calibrated polystyrene beads were used for scatter calibration and threshold adjustment according to the manufacturer's instructions. Negative controls included exosomes incubated with secondary antibody alone and isotype‐matched controls. Data were processed using NanoFCM analysis software, and CAV1‐positive exosomes were quantified as the percentage of fluorescence‐positive events relative to total exosome events after background subtraction.

### 4.11 Exos‐IP

Exosomes isolated from genetically modified HN12 cells were lysed in IP buffer (Thermo Fisher, Cat# 87787) supplemented with protease and phosphatase inhibitors. IP was performed using an anti‐CAV1 antibody or normal IgG, followed by incubation with protein A/G Sepharose beads (Amersham Biosciences, South San Francisco, CA) overnight at 4 °C on a rotating platform. After elution, protein expression was analyzed by Western blot.

### 4.12 NA Sequencing

Total RNA was extracted from HUVECs incubated with exosomes derived from *GLS1* knockdown or control HN12 cells using TRIzol (Invitrogen, Carlsbad, CA). Purified RNA samples were submitted to Novogene Corporation (Sacramento, CA) for library construction and sequencing on the Illumina HiSeq 2000 platform (RRID: SCR_020130) to generate 50 nt read length expression libraries. Independent duplicate cultures were used to minimize random variation. DEGs were identified using the DESeq R package (version 4.1.2) with the functions *estimateSizeFactors* and *nbinomTest*. KEGG pathway enrichment analysis was performed on DEGs meeting the criteria |log_2_FC|≥1 and *p* < 0.05. The RNA‐seq data generated in this study has been deposited in the Gene Expression Omnibus (GEO) under accession number GSE311744.

### 4.13 Animal Study and IHC

Six‐week‐old male NSG and C57BL/6 mice were purchased from the Jackson Laboratory (Bar Harbor, Cat# 005557). All animal experiments were approved by the Institutional Animal Care and Use Committee (IACUC) of Emory University. To evaluate GLS1 function, 5 × 10^5^ *GLS1* knockdown or control HN12 cells were suspended in 50 µL PBS/Matrigel (3:1) and injected into the buccal mucosa of NSG mice. Similarly, 5 × 10^5^ *Gls* knockdown or control MOC2 cells were injected in the same manner into C57BL/6 mice. To determine the therapeutic efficacy of CB‐839, 10 days after HN12 cell inoculation, NSG mice were randomly assigned to receive vehicle or CB‐839. CB‐839 was administered by oral gavage twice a day at a dose of 200 mg/kg body weight for 18 days. For in vivo exosome add‐back experiments, intramucosal tumors established from *GLS1* knockdown HN12 cells (2 weeks after cell inoculation) received intratumoral injections of PBS or exosomes isolated from *GLS1* knockdown or control HN12 cells, or *GLS1* knockdown HN12 cells ectopically overexpressing CAV1. Exosomes (100 µg) were resuspended in 20 µl of PBS and loaded into a Hamilton syringe fitted with a 28‐gauge needle. The needle was inserted into the center of the tumor, and the suspension was injected slowly over 60 s. To minimize reflux, the needle was retained in place for an additional 30 s prior to withdrawal. Intratumoral injections were administered once every six days for a total of three doses. Tumor dimensions were serially measured with electronic calipers, and tumor volume was calculated by the formula of V = length × width^2^ × 1/2. Body weight and physical activity of each animal were followed as markers of toxicity. Afterward, mice were sacrificed on Day 28, and tumors were excised for IHC with anti‐CD31 antibody. CD31‐positive microvessels were counted in at least six randomly selected high‐power fields per tumor section. A single microvessel was defined as any endothelial cell or cluster of cells positive for CD31, clearly separated from adjacent structures.

### 4.14 PDO‐HUVEC 3D Co‐Culture

A PDO‐HUVEC 3D system was established with modifications based on a previously described procedure [40]. Briefly, 1 × 10^3^ PDO‐derived cells were suspended with 50 µl Matrigel and seeded into 3D agarose cast (MilliporeSigma, Cat# Z764051), followed by culture for five days. Cultures were then treated with or without 5 µm CB‐839 or 20 µm GW4869 for two days. Separately, 1 × 10^3^ HUVECs were labeled with CellTracker Green BODIPY (Thermo Fisher, Cat# 2102) and subsequently added to the agarose casts containing PDOs. After 24 h, the constructs were overlaid with 2% Type I collagen (MilliporeSigma, Cat# 08–115) and maintained in culture for an additional two days. HUVEC invasion was imaged using a fluorescence microscope, and migration areas were quantified using ImageJ software (NIH).

### 4.15 Statistical Analysis

Statistical testing was performed using GraphPad Prism 9 (San Diego, CA). Experimental values are expressed as mean ± standard deviation (SD). The differences between the two groups were analyzed by an unpaired Student's *t*‐test, while one‐way analysis of variance (ANOVA) was performed to analyze differences among 3 or more groups. *p* < 0.05 was considered statistically significant.

## Author Contributions

Conceptualization and funding acquisition were carried out by Y.T. The original draft of the manuscript was written by J.Y. and Y.T., while review and editing were performed by Y.Y., N.F.S., and Y.T. The conduct of studies was undertaken by J.Y., Z.F., F.C., F.Y., S.V.K., Y.Z., and Y.L. Formal analysis was conducted by J.Y., F.C., F.Y., and Y.Z. All authors reviewed the paper and approved the final version.

## Funding

This work was partially supported by NIH/NIDCR under award numbers R01DE033433 and R01DE033691 (Y.T.). Additional support to Y.T. was provided by the Georgia CORE Cancer Research Fund, I^3^ Morningside Center Research Award, and I^3^ Nexus Research Award from Emory School of Medicine, a gift from Woodruff Fund Inc., and through the Georgia CTSA NIH award (UL1‐TR002378). The study was also supported by the Winship Invest$ Team Science Award, the Winship Invest$ Pilot Award, and the CMB Pilot Award under award number P30CA138292. Y.T. is the inaugural recipient of the Wally Award from Winship Cancer Institute.

## Conflicts of Interest

N.F.S. reports compensated and uncompensated advisory roles with: Astra Zeneca, Eisai Medical, Exelixis, Merck, Merck EMD Serono, Pfizer, Kura, Vaccinex, CUE, BionTech, GSK, TOSK, Seagen, Flamingo, Infinity, Inovio, Aveo, Medscape, Onclive, Uptodate, BMS, Cornerstone, Celldex, Surface Oncology, Astex, Imugene, Faron Pharmaceutical, Coherus, Adagene, Fulgent Springer, Nanobiotix, Taiho; funding from: Exelixis, BMS. NCS reports compensated advisory roles with GeoVax, Regeneron, Johnson & Johnson, Sensorion, Asparago Labs, and Synergy Research, in addition to research funding from Taiho Oncology. The authors declare that they have no conflicts of interest.

## Supporting information




**Supporting File**: advs75510‐sup‐0001‐SuppMat.docx.

## Data Availability

The data that support the findings of this study are available from the corresponding author upon reasonable request.
