# Supplementary material for: GLS1 Orchestrates Exosome‐Mediated Tumor‐Endothelial Communication to Facilitate Angiogenesis
Source: Adv Sci (Weinh). 2026 May 3;13(41):e75510. doi: 10.1002/advs.75510 (PMC13335626; doi:10.1002/advs.75510)
Supplement: Supplementary file 1 — Supporting File: advs75510‐sup‐0001‐SuppMat.docx. [file ADVS-13-e75510-s001.docx]

**Supporting Information**

**GLS1 orchestrates exosome-mediated tumor-endothelial communication to facilitate angiogenesis** Jianqiang Yang^1†^, Zhenzhen Fu^1†^, Fanghui Chen^1^, Soumya Vijaya Kumar^1^, Fan Yang^1^, Yaochao Zheng^2^, Yunqi Li^1^, Yao Yao^2^, Nabil F. Saba^1^, Yong Teng^1,3*^

***Correspondence:** Yong Teng, PhD, Department of Hematology and Medical Oncology

Emory University, 201 Dowman Dr, Atlanta, GA 30322

Tel: (404)712-8514; E-mail: [yong.teng@emory.edu](mailto:yong.teng@emory.edu)

**This file includes**

**Supplementary Figures S1-S12**

**Supplementary Tables S1-S3**


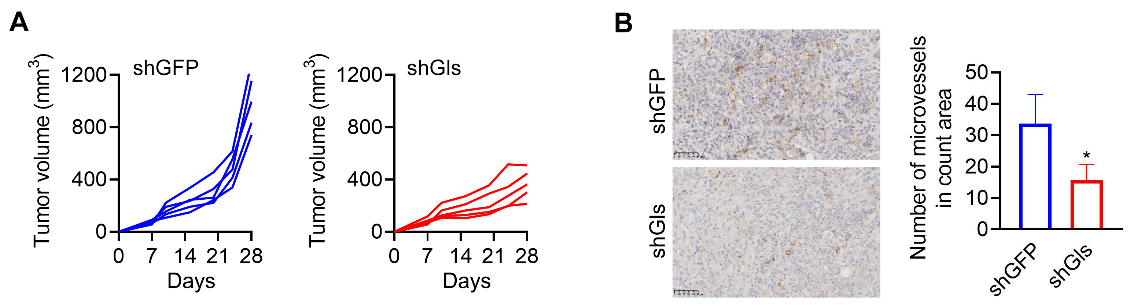


**Figure S1. Knockdown of *Gls* inhibits angiogenesis in the immunocompetent mice.** (**A**) Effect of *Gls* knockdown on MOC2 tumor growth in C57BL/6 mice (n = 5 mice/ group). *Gls* knockdown (shGls) or control (shGFP) MOC2 cells were injected into the buccal mucosa of C57BL/6 mice under anesthesia. The experiment was terminated 28 days after cell inoculation. (**B**) IHC of tumor tissues derived from *Gls* knockdown or control MOC2 xenografts using anti-CD31 antibody. Quantitative data (n = 5 mice/ group) are presented in the right panel. **p*<0.05.


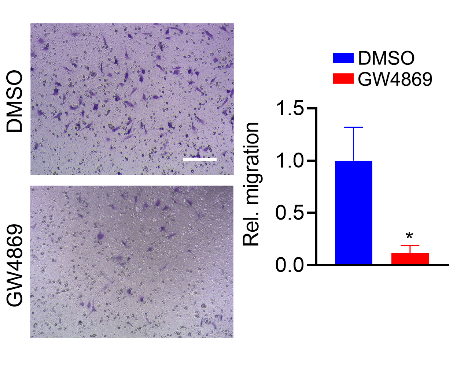


**Figure S2. HUVEC migration following a 24-hour transwell incubation with conditioned media derived from HN12 cells treated with or without 20 µM GW4869.** Scale bar: 50 μm. **p*<0.05.


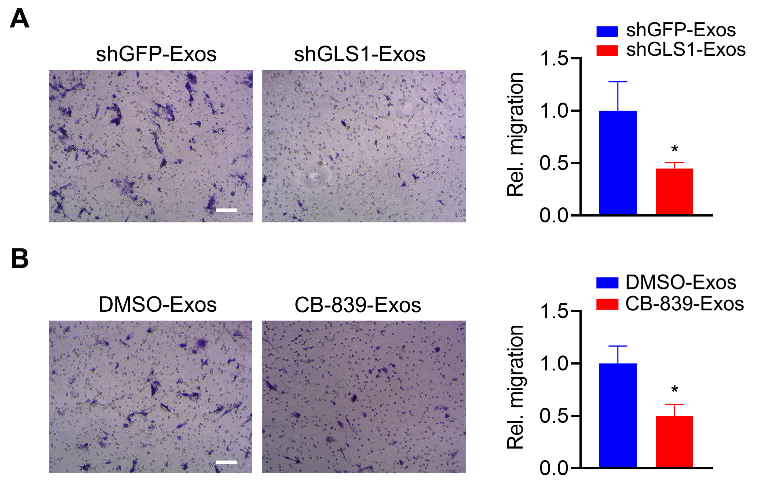


**Figure S3. Exosomes from *GLS1* knockdown HN6 cells impair HUVEC migration.** (**A**) HUVEC migration following a 24-hour transwell incubation with exosomes from *GLS1* knockdown or control HN6 cells. (**B**) HUVEC migration following a 24-hour transwell incubation with exosomes from HN6 cells treated with CB-839 or DMSO. In (**A, B**), representative images and quantitative data (n=3) are shown in the left and right panels, respectively. Scale bar: 50 μm. **p*<0.05.


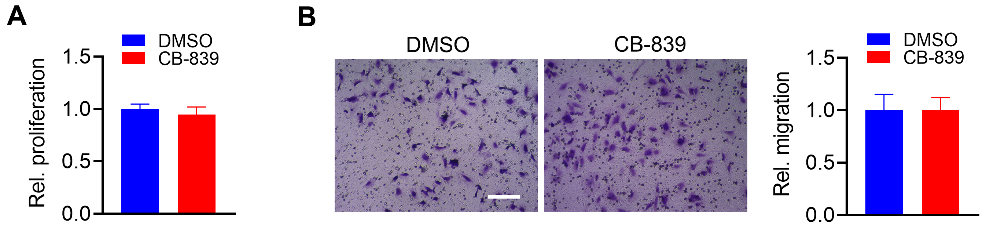


**Figure S4. CB-839 has no effect on HUVEC proliferation and migration.** (**A**) Effect of 2 µM CB-839 on HUVEC cell proliferation following a 72-hour treatment. (**B**) Effect of 2 µM CB-839 on HUVEC cell migration following a 24-hour transwell incubation. Representative images and quantitative data (n=3) are shown in the left and right panels, respectively. Scale bar: 50 μm.


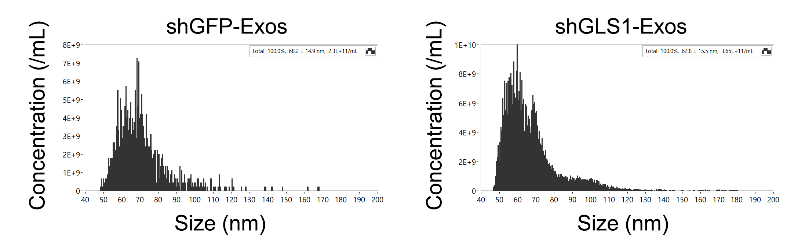


**Figure S5. Nanoparticle tracking analysis showing the size distribution profiles of exosomes derived from *GLS1* knockdown and control HN12 cells.**


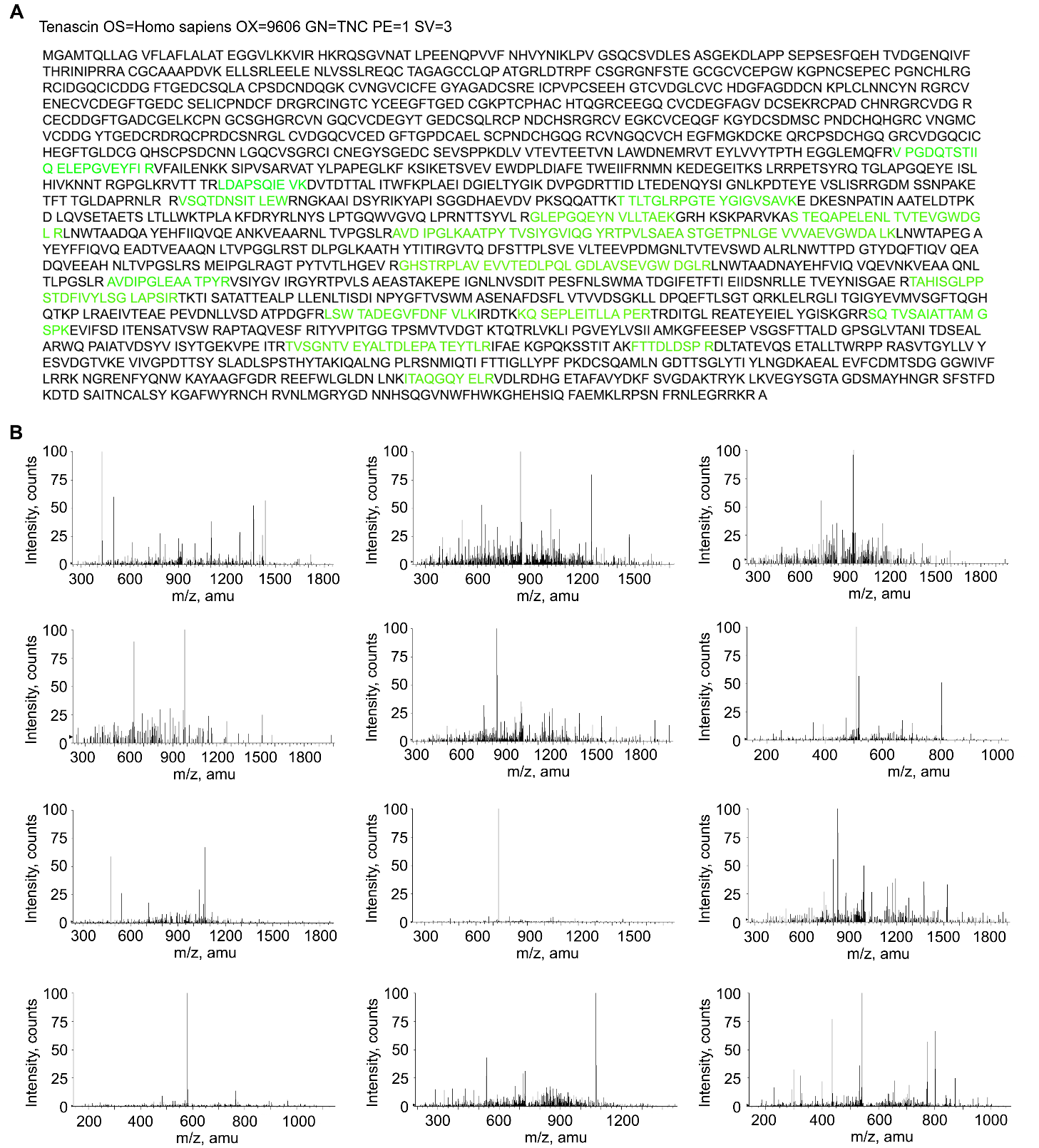


**Figure S6. Amino acid sequences (A) and representative mass spectrograms (B) of tryptic TNC peptides identified from HN12 cell-derived exosomes by LC-MS analysis.**


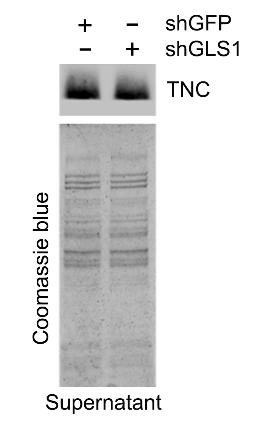


**Figure S7. Knockdown of *GLS1* does not affect TNC section from HNSCC cells.** Effect of *GLS1* knockdown on TNC protein levels in exosome-depleted HN12 cell supernatant determined by Western blot. Coomassie staining was used as a loading control. In this experiment, exosome-depleted supernatant was prepared by initially centrifuging the culture medium at 3,000 × g for 20 min at 4°C to remove cellular debris, followed by ultracentrifugation at 100,000 × g for 120 min at 4°C to pellet exosomes.


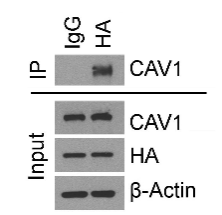


**Figure S8.** **Interaction between USP1 and CAV1 in 293T cells expressing HA-USP1.** HA-USP1-expressing 293T cell lysates were immunoprecipitated with anti-HA antibody, followed by Western blot analysis using anti-CAV1 antibody. Pre-immune IgG was used as a negative control.


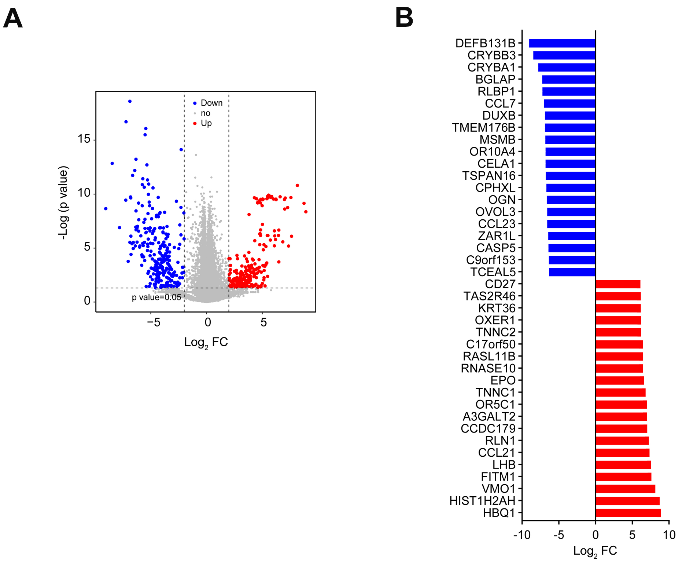


**Figure S9. RNA-seq analysis of HUVECs co-cultured with exosomes derived from *GLS1* knockdown and control HN12 cells.** (**A**) Volcano plot showing differentially expressed genes (DEGs) in HUVECs co-cultured with exosomes from *GLS1* knockdown versus control HN12 cells. (**B**) Top 20 upregulated and downregulated DEGs in HUVECs co-cultured with exosomes from *GLS1* knockdown HN12 cells compared with those co-cultured with exosomes from knockdown control HN12 cells.


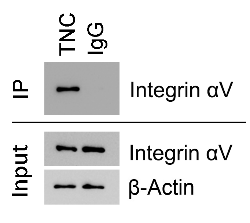


**Figure S10. TNC interacts with integrin αV in HUVECs.** IP analysis of the interaction between TNC and integrin αV in HUVECs. HUVEC cell lysates were immunoprecipitated with anti-TNC antibody, followed by Western blot analysis using anti-integrin αV antibody. Preimmune IgG served as a negative control.


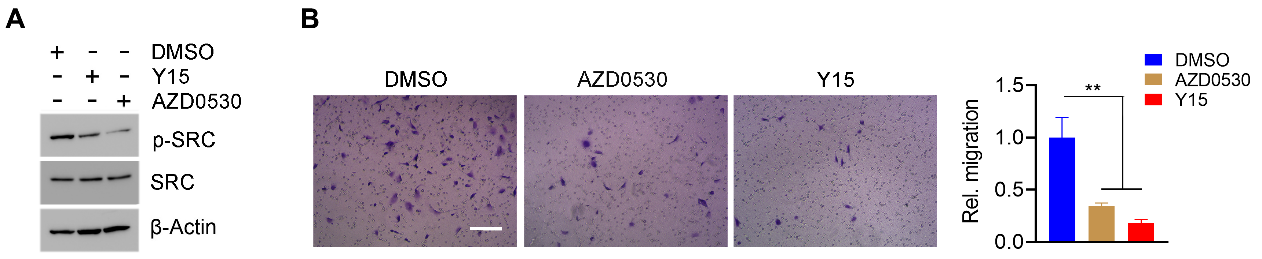


**Figure S11. Blocking FAK-SRC signaling impairs HUVEC migration.** (**A**) Total and phosphorylated SRC protein levels in HUVECs following the treatment with 10 µM AZD0530 or Y15 for 24 h. (**B**) HUVEC migration following the treatment with 10 µM AZD0530 or Y15 for 24 h. Representative images and quantitative data (n=3) are shown in the left and right panels, respectively. Scale bar: 50 μm. ***p*<0.01.


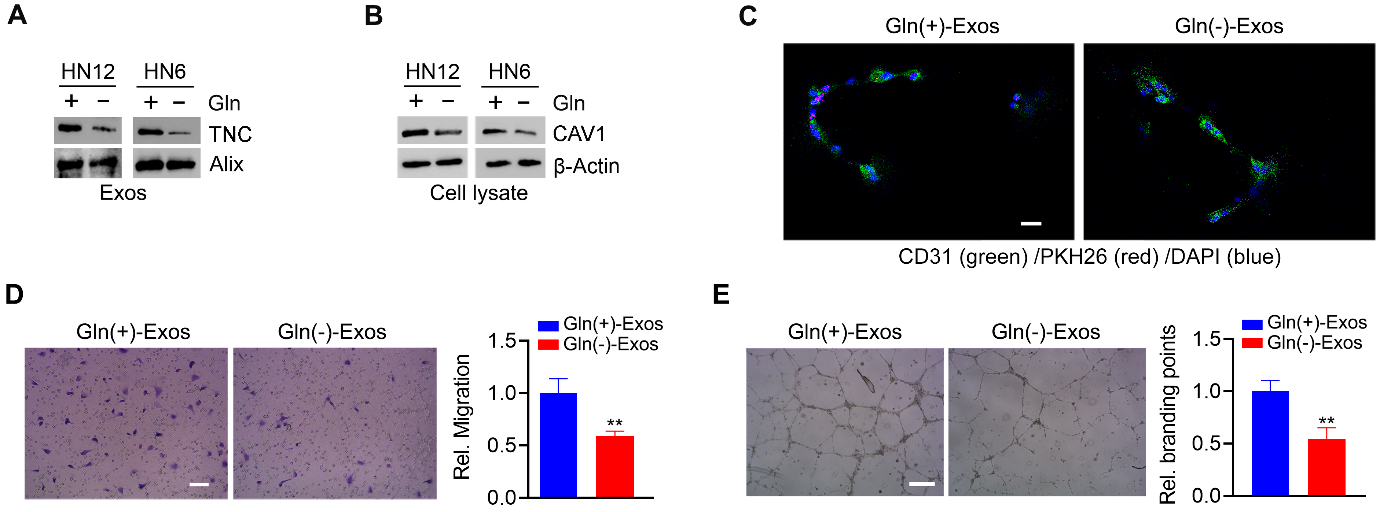


**Figure S12. Glutamine deprivation in HNSCC cells suppresses CAV1-TNC signaling, leading to decreased HUVEC migration and tube formation.** (**A**) Effect of glutamine deprivation on TNC protein levels in HN6 and HN12 cell exosomes determined by Western blot. (**B**) Effect of glutamine deprivation on CAV1 protein levels in HN6 and HN12 cells determined by Western blot. (**C**) Confocal microscopy images showing HUVEC uptake of exosomes derived from HN12 cells with or without glutamine deprivation for 24 h. HUVECs were stained with anti-CD31 antibody (green), exosome membranes were labeled with PKH26 (red), and nuclei were counterstained with DAPI (blue). Scale bar: 50 μm. (**D**) HUVEC migration following a 24-hour transwell incubation with exosomes derived from HN12 cells with or without glutamine deprivation. (**E**) HUVEC tube formation following a 24-hour co-culture with exosomes derived from HN12 cells with or without glutamine deprivation. In (**D, E**), representative images and quantitative data (n=3) are shown in the left and right panels, respectively. Scale bar: 50 μm. ***p*<0.01.

**Table S1. Proteomic analysis identified proteins that are absent in *GLS1* knockdown HN12 cells compared with control cells**

| **Protein** | **Molecular weight** | **Protein coverage** | | |
| --- | --- | --- | --- | --- |
| TNC | 240700.15 Da | 2201 AA (total) | 320 AA (covered) | 14.54% |
| ARPC2 | 34311.49 Da | 300 AA (total) | 39 AA (covered) | 13.00% |
| CCT5 | 59632.81 Da | 541 AA (total) | 46 AA (covered) | 8.50% |
| HYOU1 | 111266.21 Da | 999 AA (total) | 79 AA (covered) | 7.91% |
| CLDN1 | 22728.58 Da | 211 AA (total) | 16 AA (covered) | 7.58% |
| PLSCR3 | 31627.95 Da | 295 AA (total) | 20 AA (covered) | 6.78% |
| CCT8 | 59582.51 Da | 548 AA (total) | 37 AA (covered) | 6.75% |
| PLBD2 | 65430 Da | 589 AA (total) | 37 AA (covered) | 6.28% |
| CFH | 139004.7 Da | 1231 AA (total) | 39AA (covered) | 3.17% |

**Note:** Only proteins with a protein coverage greater than 3%, as determined by LC-MS analysis, are included in this table. Lower coverage proteins were excluded to minimize potential false positives.

**Table S2. Antibodies used in this study**

| **Antibodies** | **Application** | **Source** | **Identifier** |
| --- | --- | --- | --- |
| Glutaminase-1/GLS1 (E9H6H) XP® Rabbit mAb, | WB | Cell Signaling Technology | Cat# 56750 |
| MONOCLONAL ANTI-B-ACTIN, CLONE AC-74 | WB | MilliporeSigma | Cat# A5316 |
| Tenascin C (E5J3B) Rabbit mAb | WB, IF | Cell Signaling Technology | Cat# 33352 |
| HA-Tag (C29F4) Rabbit mAb | WB, IP | Cell Signaling Technology | Cat# 3724 |
| USP1 (D37B4) Rabbit mAb | WB, IP | Cell Signaling Technology | Cat# 8033 |
| CD31 (PECAM-1) Monoclonal Antibody (390) | IF, IHC | ThermoFisher | Cat# 14-0311-85 |
| [CD31 (PECAM-1) Monoclonal Antibody (7O7K3)](https://www.thermofisher.com/antibody/product/CD31-PECAM-1-Antibody-clone-7O7K3-Recombinant-Monoclonal/MA5-37858) | IHC | ThermoFisher | Cat # MA5-37858 |
| Caveolin-1 (D46G3) XP® Rabbit mAb | WB, IP, IF, Exosome-Flow | Cell Signaling Technology | Cat# 3267 |
| Alix (E6P9B) Rabbit mAb | WB | Cell Signaling Technology | Cat# 92880 |
| Calnexin (C5C9) Rabbit mAb | WB | Cell Signaling Technology | Cat# 2679 |
| CD9 (D8O1A) Rabbit mAb | WB | Cell Signaling Technology | Cat# #13174 |
| HSP70 (D69) Antibody | WB | Cell Signaling Technology | Cat# 4876 |
| Phospho-Src (Tyr530) Antibody | WB | Cell Signaling Technology | Cat# 2105 |
| Src (36D10) Rabbit mAb | WB | Cell Signaling Technology | Cat# 2109 |
| Phospho-FAK (Tyr397) Antibody | WB | Cell Signaling Technology | Cat# 3283 |
| FAK (D2R2E) Rabbit mAb | WB | Cell Signaling Technology | Cat# 13009 |
| Integrin alpha V (ITGAV/CD51) Rabbit mAb | WB, IP | Abclonal | Cat# A19071 |
| Goat anti-Rabbit IgG (H+L) Cross-Adsorbed Secondary Antibody, Alexa Fluor™ 488 | IF, Exosome-Flow | ThermoFisher | Cat# A-11008 |
| Anti-rabbit IgG, HRP-linked Antibody | WB | Cell Signaling Technology | Cat# 7074 |
| Anti-mouse IgG, HRP-linked Antibody | WB | Cell Signaling Technology | Cat# 7076 |

**Table S3. Primers used in this study**

| **Primers** | **Sequence** | **Application** |
| --- | --- | --- |
| TNC-F | CATGGTCATAGCTGTTTCC | Subclone |
| TNC-R | TCGAGGTCGACGGTATC | Subclone |
| ITGA2-F | TTGCGTGTGGACATCAGTCTGG | RT-qPCR |
| ITGA2-R | GCTGGTATTTGTCGGACATCTAG | RT-qPCR |
| ITGB8-F | CTGTTTGCAGTGGTCGAGGAGT | RT-qPCR |
| ITGB8-R | TGCCTGCTTCACACTCTCCATG | RT-qPCR |
| ITGAV-F | AGGAGAAGGTGCCTACGAAGCT | RT-qPCR |
| ITGAV-R | GCACAGGAAAGTCTTGCTAAGGC | RT-qPCR |
| β-actin-F | CTACAATGAGCTGCGTGTG | RT-qPCR |
| β-actin-R | TGGGGTGTTGAAGGTCTC | RT-qPCR |
